# Physiological insights into electrodiffusive maintenance of gastric mucus through sensitivity analysis and simulations

**DOI:** 10.1007/s00285-021-01643-w

**Published:** 2021-08-26

**Authors:** Manu Aggarwal, N. G. Cogan, Owen L. Lewis

**Affiliations:** National Institute of Health; Florida State University; University of New Mexico

**Keywords:** Gastric Mucus, Physiological gels, Electrodiffusion, Sobol’ sensitivity

## Abstract

It is generally accepted that the gastric mucosa and adjacent mucus layer are critical in the maintenance of a pH gradient from stomach lumen to stomach wall, protecting the mucosa from the acidic environment of the lumen and preventing auto-digestion of the epithelial layer. No conclusive study has shown precisely which physical, chemical, and regulatory mechanisms are responsible for maintaining this gradient. However, experimental work and modeling efforts have suggested that concentration dependent ion-exchange at the epithelial wall, together with hydrogen ion/mucus network binding, may produce the enormous pH gradients seen *in vivo*. As of yet, there has been no exhaustive study of how sensitive these modeling results are with respect to variation in model parameters, nor how sensitive such a regulatory mechanism may be to variation in physical/biological parameters. In this work, we perform sensitivity analysis (using Sobol’ Indices) on a previously reported model of gastric pH gradient maintenance. We quantify the sensitivity of mucosal wall pH (as a proxy for epithelial health) to variations in biologically relevant parameters and illustrate how variations in these parameters affects the distribution of the measured pH values. In all parameter regimes, we see that the rate of cation/hydrogen exchange at the epithelial wall is the dominant parameter/effect with regards to variation in mucosal pH. By careful sensitivity analysis, we also investigate two different regimes representing high and low hydrogen secretion with different physiological interpretations. By complementing mechanistic modeling and biological hypotheses testing with parametric sensitivity analysis we are able to conclude which biological processes must be tightly regulated in order to robustly maintain the pH values necessary for healthy function of the stomach.

## 1 Introduction

The surface of the gastric epithelium is covered in a continuous layer of mucus, an entangled, hydrated gel of polymeric proteins. For more than six decades, the physiology community has accepted that this gastric mucus layer provides a protective barrier, shielding the gastric wall (mucosa) from the stomach interior (lumen). In addition to preventing epithelial infection by most bacteria and other pathogens, the gastric mucus layer appears to protect the mucosa from the high acidity and digestive enzymes within the gastric cavity, preventing auto-digestion of the epithelium [1, 2]. In numerous mammalian species, the gastric mucus layer has been observed to support a massive (10^5^-fold) hydrogen gradient across a relatively small length scale (several hundred μm) [3–5]. Failure to maintain this pH gradient across the layer (due to infection or misregulation) is associated with numerous pathologies, including gastric ulcers and cancer [6].

There are numerous unanswered questions regarding the mechanisms that give rise to the protective function of the mucus layer. Competing hypotheses exist regarding how acid (which is produced within crypts on the epithelial surface) is transported to the stomach lumen without acidifying the space immediately adjacent to the epithelium [5, 7, 8]. Unfortunately, the experimental evidence is inconclusive, and no consensus has been reached [9]. It has been known for some time that the the gastric epithelium also secretes bicarbonate, which can neutralize dissolved hydrogen ions and contributes to the protective nature of the mucus layer [10, 11]. However, there has been some debate if hydrogen, once transported to the stomach lumen, diffuses normally back down its own concentration gradient towards the epithelium, and if not, what mechanisms retard its diffusive flux [12]. Finally, there is no clear understanding of how the secretory processes within the epithelium are coordinated with the physical processes governing molecular diffusion through the mucus layer in order to maintain healthy gastric function. In this regard, mathematical models provide an invaluable tool, allowing one to analyze the interplay of physical and biological processes that give rise to the gastric pH gradient and often providing insights that may be inaccessible by experimental techniques.

It is known that the ionic composition of gastric secretions can vary in response to numerous stimuli, including histamine levels, the recency of a meal, and the contents of the gastric lumen [10, 13, 14]. At least one theoretical study has analyzed a model of how acid secretion is controlled by nervous and endocrine stimuli [15]. However, this model lacks any description of spatial variation in hydrogen concentration and therefore cannot explain the hydrogen gradient seen *in vivo*. Other, more physical, models have attempted to quantify the rate of hydrogen flux through gastric mucus and use this to calculate the secretion of bicarbonate required to maintain a healthy pH gradient [16]. However, experiments attempting to measure rates of bicarbonate secretion disagree with the model predictions [17].

To date, few modeling works have attempted to couple a physical description of diffusion through the mucus layer to a biologically informed model of secretion at the epithelial surface. One such model was put forth by Lewis, et. al in [18]. The underlying assumption made in this model is that hydrogen is transported from epithelium to lumen sequestered on mucin polymers (and therefore is not a dissolved, diffusive species during this process), and then released in the lumen by auto-digestion of the mucus network, as suggested by Schreiber, et. al [5]. A physical model of electro-diffusion through a complex gel-like material was coupled with non-linear boundary conditions that mimic the anti-port ion exchange proteins expressed by gastric epithelial cells [11]. In particular, bicarbonate secretion in this model functionally depends on the ionic concentration (chloride, specifically) in the space immediately adjacent to the epithelial surface. Analysis showed that such a model was capable of reproducing the pH gradient across the mucus layer observed *in vivo.* In fact, the pH gradient was maintained regardless of nearly all variations in model parameters, with one exception: knocking out the hydrogen/sodium exchange proteins in the epithelial surface (by setting a parameter to zero) destroyed or even inverted the pH gradient across the epithelial surface and caused the predicted wall pH to depend “strongly” on the other model parameters.

The sensitivity of model predictions to variation in parameters is an important issue in the realm of biological modeling. Lack of accuracy or precision in experimental measurements can lead to uncertainty in model parameter values. Quantitative assessment of the impact that this uncertainty has on model predictions is therefore a valuable tool. Furthermore, the physical quantities which model parameters represent may not be fixed in the biological system of interest (varying temporally, for example). The sensitivity (or insensitivity) of model output to variations in these values gives meaningful insight into the underlying biology. The analysis of [18] represents what is often termed “one-at-a-time” sampling in the field of sensitivity analysis: restriction to a lower dimensional hyperplane of parameter space. Not only do such lower-dimensional analyses risk missing important features of the system behavior, in some cases they may even lead to misleading results [19]. Because of this, we will analyize a crucial prediction of the mathematical model from [18] using a more robust, global measure of sensitivity.

In this work, we use Sobol’ Indices (SIs) to perform a global sensitivity analysis of the model of gastric epithelial pH regulation first put forth in [18]. In Section 2, we outline the mathematical model as well as the relevant parameters. Section 3 provides a discussion of the theory and calculation of SI. In Section 4, we report the SI calculated for the model parameters and discuss the dependence of epithelial pH distribution on various parameters. In particular, we see that the rates at which ion exchange events take place at the epithelial surface exert the strongest control over the distribution of epithelial pH, followed by the strength of hydrogen secretion. To further explore the dependence of mucosal pH on these “important” parameters, we perform several restricted Sobol’ analyses where specific parameters are fixed at extremal values. These parameter regimes replicate physiologically relevant situations (stimulated acid secretion, for example). The functional dependence of wall pH distribution on the remaining parameters is quantified, and their SI are calculated. Finally, we discuss the physiological significance of these results in Section 5.

## 2 Mathematical Model

Here, we utilize a mathematical description of diffusion through a gastric mucus layer that was first put forth in [18]. The model was developed based on the experiments of Schreiber *et al.* to mathematically investigate the proposed mechanism of hydrogen transport proposed in [5]. To our knowledge this represents the only physics-based model of hydrogen diffusion through mucus which has successful predicted the enormous pH gradient (from lumen to stomach wall) observed in mammalian stomachs. It is based upon the Nernst-Planck equations of electrodiffusion in conjuction with a “two-phase gel” representation of mucus. The two-phase gel framework has been used extensively to model gel-like substances in biological contexts [20, 21]. At each point in space there simultaneously exist two materials: the “gel” (cross-linked mucin polymer network), and the “solvent” (interstitial hydrating fluid). The local composition of the combined material is described by the dimensionless quantities *θ*_*s*_ and *θ*_*g*_ ≔ 1 − *θ*_*s*_ which represent the “volume fraction” of solvent and network, respectively (i.e. the proportion of local volume that is made up of each constituent material). In this work, we are primarily concerned with the diffusion of ionic species dissolved within the solvent phase. Therefore, the phases *θ*_*s*_ and *θ*_*g*_) will be fixed in time, and their spatial profiles specified to represent the inhomogeneous material through which ions are moving (discussed at more length below).

The state variables of the model are the concentration of four ionic species and the electric potential gradient within the domain. Because the ions are dissolved in (and diffuse through) only the solvent phase of the gel, we define the concentration variables as moles per volume of *solvent*, not moles per total volume. Hydrogen and bicarbonate are denoted *C*_H_ and *C*_B_, both with units of moles per liter (Molar). All other cations and anions are represented by *C*_I_ and *C*_A_, respectively, measured in units of Equivalent per liter (which we denote M for notational convenience). The most common ions in the gastric interior are sodium (cations), potassium (cations), and chloride (anions) [22]. By including sodium and potassium in a single variable, we assume that both cations have the same chemical properties and diffusion constants. The spatial variable *x* measures distance from the epithelial wall of the stomach towards the lumen, and thus the location *x* = 0 corresponds to the epithelial surface. The concentration of ionic species is governed by a Nernst-Planck-like equation, which accounts for the flux of ions due to advection, diffusion, and electric potential gradients, as well as any reactions and sources that impact ionic concentration. A time-dependent version of this model was used in [18], and a careful derivation may be found in [23], however the present work is only concerned with steady state behavior. The equation which governs each ionic species may be described as the balance of fluxes and sources:

(1) $$\frac{\partial }{\partial x}\left(\underset{\text{I}}{\underset{︸}{u\left(x\right)C_{j}}}\right)=\frac{1}{\theta_{s}\left(x\right)}\frac{\partial }{\partial x}\left(\theta_{s}\left(x\right)\left(\underset{\text{II}}{\underset{︸}{D_{j}\frac{\partial }{\partial x}C_{j}}}+\underset{\text{III}}{\underset{︸}{z_{j}D_{j}C_{j}\frac{\partial }{\partial x}\Psi }}\right)\right)+\underset{\text{IV}}{\underset{︸}{g_{j}}},$$

Here, the index *j* may take the values *H, B, A*, or *I* for hydrogen, bicarbonate, anions, and cations respectively. Terms I, II, and III represent fluxes of ionic concentration due to advective, diffusive, and electric effects repsectively. Term IV captures any reactions and/or sources that impact the concentration of species *j*. The quantity *u*(*x*) is the velocity of the fluid solvent (in cm/sec), which results from secretion of fluid at the stomach wall. The parameters *D_j_* and *z_j_* are the diffusion coefficient (cm^2^/sec) and valence (±1) of species *j* respectively. The variable *Ψ* is the non-dimensional electric potential (which may be converted to voltage by multiplying by *RT/F*, where *R* is the ideal gas constant, *T* is absolute temperature, and *F* is the Faraday constant). Note that Eq. (1) is similar to a standard steady state Nernst-Planck equation, with the exception of two appearances of *θ*_*s*_(*x*) on the right hand side. These terms account for the fact that the ionic species are transported within the *solvent* phase of a mixture whose composition varies spatially (see [23]). The electric potential *Ψ* is determined by enforcing electro-neutrality, which is the principle that ionic concentrations may not result in a net charge at any location within space. Mathematically, this is expressed as

(2) $$\underset{j}{\sum }z_{j}C_{j}=0.$$

Thus, *Ψ* does not have its own equation but may be viewed as a Lagrange multiplier enforcing the constraint Eq. (2) on the set of evolution equations given by Eq. (1). We note here that the electroneutrality constraint (Eq. (2)) does not account for any charge on the mucus network itself. Though mucus polymers carry negative charge groups, the work of Schreiber, *et al*. indicates that *in vivo* mucus carries a large concentration of bound hydrogen ions, effectively neutralizing many of these negative charge groups [5]. Thus, the model implicitly assumes that approximately all of the negative charge on mucus is neutralized by bound cations.

**Fig. 1:**
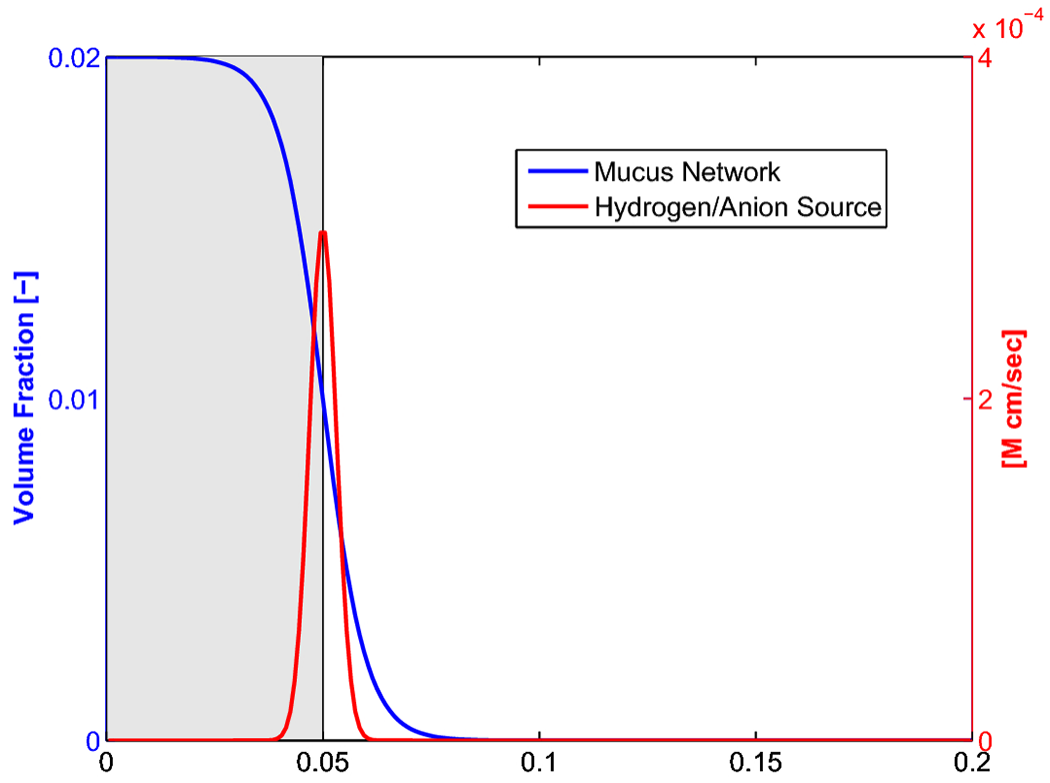
Illustration of gel volume fraction (*θ_g_*) and Hydrogen/Anion source profiles. Recall that *θ_s_* = 1 – *θ_g_*.

Other works have explored the dynamic rearrangement of mucus in response to ionic diffusion using a similar modeling framework [24]. However, as we wish to focus on the steady state behavior of ionic species, we will treat both the solvent velocity and volume fraction as given model inputs. We assume that gastric mucus is constantly produced at the epithelial surface, moves away from the wall, and is degraded as it approaches the lumen in a manner that maintains a time-independent profile *θ*_*g*_(*x*). We choose *θ*_*g*_(*x*) to have a spatial profile that reflects a “standing front” defining the transition from the mucus gel layer to the stomach lumen. Figure 1 shows the spatial form of *θ*_*g*_(*x*) (recall that *θ*_*s*_(*x*) = 1 — *θ*_*g*_(*x*)). The transition is located 500 μm away from the wall, and the mucus layer is indicated by the grey shaded region in Fig. 1. We define the “mucus layer” as the region where mucus network occupies more than than 1% of the local volume (i.e *θ*_*g*_ > 0.01, *θ*_*s*_ < 0.99). The “lumen” is therefore the region where *θ*_*g*_ < 0.01 and *θ*_*s*_ > 0.99. We note here that this model does not explicitly describe the swelling of mucus that happens on very short length and time scales immediately after secretion, nor does it incorporate the (relatively modest) changes in mucus volume fraction which may result from changes in monovalent cation concentration (as all ions in the model are monovalent) [25, 26]. Finally, the profile of *u*(*x*) is chosen to ensure that the quantity *u*(*x*)*θ*_*s*_(*x*) is constant in space (as required by conservation of solvent mass) and approximates observed rates of fluid secretion from the gastric mucusa (see [18]). Choosing *u*(*x*) in this manner also ensures that Eq. (1) (in the absence of sources and reactions) is conservative in the quantities *θ*_*s*_(*x*)*C_j_*, which are the total ionic concentrations (moles per *total* volume).

The terms *g_j_* incorporate any sources or chemical reactions that may impact the four concentrations. Hydrogen is affected by the buffering reaction with bicarbonate (which proceeds according to the law of mass action). Because the dissociation constant of hydrogen and bicarbonate is small (4.2 × 10^−7^ M [16]), we assume that this reaction is non-reversible. In [5], Schreiber et. al proposed a mechanism of hydrogen transport in which hydrogen ions are also produced by a source located at the edge of the mucus layer. Mathematically, we represent this as a source *S*(*x*) supported in the same region where the volume fraction transitions from mucus layer to lumen. This is to model the release of hydrogen due to enzymatic degradation of the mucus gel itself, in accordance with the mechanism proposed by Schreiber et. al [5]. Because the gel layer is fixed, the source of hydrogen is assumed to be independent of time, but localized spatially,

(3) $$g_{\text{H}}\text{ = }S\text{(}x\text{) - }kC_{\text{H}}C_{\text{B}}.$$

In [18], a hydrogen source was used that represents the “average” rate of hydrogen secretion in the gastric mucosa. In this work, we investigate the effects of changes in hydrogen secretion on model predictions, and therefore our source is given by $S(x)=S_{0}\hat{S}(x)$, where $\hat{S}(x)$ is the “baseline” hydrogen source used in [18]. The constant *S*_0_ is a parameter which controls the magnitude of hydrogen production. The profile of $\hat{S}(x)$ is shown in Fig. 1. The concentration of bicarbonate is affected by the same hydrogen/bicarbonate buffering reaction, hence

(4) $$g_{\text{B}}\text{ = - }kC_{\text{H}}C_{\text{B}}.$$

Electro-neutrality implies that any hydrogen ion released by mucus degradation at the interface must be accompanied by a corresponding negatively charged particle. Therefore, the source term also affects the concentration of anions:

(5) $$g_{\text{A}}\text{ = }S\text{(}x\text{)}.$$

Finally, we assume that cations are not affected by any sources or reactions:

(6) $$g_{\text{I}}=0.$$

Equation (1) must be accompanied by boundary conditions at the wall (*x* = 0) and the lumen interior (*x* = *L*). At the wall (*x* = 0), we impose a flux relation that depends on the local ionic concentrations at the epithelial surface. We *do not* explicitly specify the flux of any ion through the epithelial surface. For each species, the *total* flux from the transport equation is

(7) $$\phi_{j}=-D_{j}\frac{\partial }{\partial x}C_{j}-D_{j}z_{j}C_{j}\frac{\partial }{\partial x}\Psi +C_{j}u\left(x\right).$$

When evaluated at the mucosa (*x* = 0), this expression must be equal to the flux through the ion-exchange proteins expressed by the epithelial cells that make up the mucosal wall. These epithelial cells express two ion exchange proteins that we account for here: one which exchanges bicarbonate and chloride (in a 1-to-1 ratio), and another which exchanges hydrogen and sodium (again, 1-to-1) [27]. Mathematically, our boundary conditions at the left boundary are given by

(8) $${\phi_{\text{H}}|}_{x=0}={k_{\text{HI}}\left(C_{\text{I}}-\delta_{\text{HI}}C_{\text{H}}\right)|}_{x=0},\;\;\;{\phi_{\text{I}}|}_{x=0}=-{k_{\text{HI}}\left(C_{\text{I}}-\delta_{\text{HI}}C_{\text{H}}\right)|}_{x=0},$$

(9) $${\phi_{\text{B}}|}_{x=0}={k_{\text{AB}}\left(C_{\text{A}}-\delta_{\text{AB}}C_{\text{B}}\right)|}_{x=0},\;\;\;{\phi_{\text{A}}|}_{x=0}=-{k_{\text{AB}}\left(C_{\text{A}}-\delta_{\text{AB}}C_{\text{B}}\right)|}_{x=0}.$$

The terms on the right hand side represent a simplified, linear model of flux due to anti-port ion exchange proteins [28]. Concentrations at the stomach interior (which we define as *x* = 0.2 cm) are given by Dirichlet boundary conditions.

(10) $${C_{\text{H}}|}_{x=L}=H_{L},\;{C_{\text{I}}|}_{x=L}=I_{L},$$

(11) $${C_{\text{B}}|}_{x=L}=B_{L},\;{C_{\text{A}}|}_{x=L}=A_{L}.$$

These values may not be chosen independently. Electroneutrality in the stomach interior requires that

(12) $$H_{L}\text{ + }I_{L}\text{ - }B_{L}\text{ - }A_{L}\text{ = 0}.$$

Several model parameters are either not well known, or may actively change in physiological situations. *S*_0_ represents the magnitude of hydrogen production by the gastric mucosa, which is known to change in response to physiological stimuli [14, 22, 29]. The parameters *k*_HI_ and *k*_AB_ quantify the rate at which hydrogen/sodium and bicarbonate/chloride exchange takes place. These values are presumably a function of the density of the anti-port proteins expressed by the gastric epithelium, as well as the specific thermodynamic properties of a single exchange event. These quantities are difficult to estimate, and may not remain constant *in vivo*. Similarly, *δ*_HI_ and *δ*_AB_ represent a “bias” in the respective ion exchange proteins, which can be related to the concentration of individual ionic species within the epithelial cells [18]. Existing estimates for these values are based on data for various cell types and may not necessarily be applicable to gastric epithelial cells. Finally, *H_L_, I_L_, B_L_* and *A*_L_ and represent ionic concentrations within the bulk of the stomach, which our model treats as prescribed, constant values, but which are known to vary temporally *in vivo* [13]. The one exception to this is *B_L_*, which is the lumenal concentration of bicarbonate. This value is essentially zero in all situations, and which we take to be 10^−14^ M (simply for numerical purposes). Therefore, the model has 7 parameters that may reasonably vary *in vivo*, or are poorly estimated in the literature: *S*_0_, *k*_HI_, *k*_AB_, *δ*_HI_, *δ*_AB_, *H _L_*, and *I _L_* (the electro-neutrality constraint determines *A_L_*). The main purpose of this work is to perform sensitivity analysis of the model predictions with respect to these 7 parameters. This analysis will help determine which parameters play a significant role in the model prediction of [18]. These “sensitive” parameters require accurate estimates. Conversely, “insensitive” parameters indicate values whose variations have little effect on model predictions.

To do this, we must first decide on a “Quanitity of Interest” (QoI). As our QoI, we will use the negative logarithm (base 10) of the hydrogen concentration at the left boundary

(13) $${\text{QoI = -log}}_{\text{10}}\text{ (}H_{\text{0}}{\text{)}≔-\log}_{\text{10}}\text{ (}C_{\text{H}}{|}_{x=0})=\text{pH at left boundary}.$$

This corresponds to the pH at the mucosal surface of the stomach, and can be interpreted as a measurement of gastric health (a neutral stomach wall with pH approximately 6 or 7 would indicate a healthy stomach, while significantly lower pH would indicate an unhealthy stomach). Therefore, one may view sensitivity analysis of this QoI as quantifying the sensitivity of “predicted stomach health” to variations in physiological parameters.

## 3 Sensitivity Analysis

To analyze the sensitivity of the non-linear relationship between the pH at the mucosal surface of the stomach and the seven parameters (implicitly defined by Eqs. (1) to (13)), we use Sobol’ indices (SI). In [30] Sobol’ defined and developed the theory for SI, variance-based sensitivity measures that do not assume linearity and monotonicity in the mathematical model. This makes them attractive to a large class of models across different fields, and they have since been used in engineering [31], physical systems [32, 33], biological systems [34], and economic models [35]. Moreover, simultaneous variation in all of the parameters is considered over the entire parameter space to compute SIs, providing a comprehensive exploration of the sensitivity of the output to the inputs. Hence, SIs are classified as *global* sensitivity analysis indices. The numerical methods that we used to estimate total and first-order SIs in this work, are based on Monte-Carlo estimation of integrals over a volume in the full-dimensional parameter space. As a result, it can be computationally expensive to estimate SIs if the number of parameters, or the dimensionality of the parameter space, is large [36, 37]. However, as we have previously mentioned, less computationally expensive *local* methods like one-at-a-time sampling can miss important parameter interactions, giving misleading conclusions [19]. The reader is directed to [38–40] for a review of various sensitivity measures.

### 3.1 Mathematical theory and notation

Given a scalar quantity of interest, *y*, as a square-integrable function of parameters {*p*_1_, …, *p*_*N*_}, where *p*_*i*_ ∈ [*a_i_, b_i_*],

$$y\text{ = }f\text{(}p_{\text{1}}\text{,}\ldots \text{,}p_{N}\text{) = }f\text{(}P\text{),}\;p_{i}\;\in [a_{i}\text{, }b_{i}\text{],}$$

Sobol’ showed in [30] that there exists a unique functional decomposition,

(14) $$f=f_{0}+\sum_{I\subset D}f_{I},$$

where *f*_0_ is a constant, *I* ⊂ *D* {1, …, *N*}, and *f_I_* is a function of parameters {*p_i_*} where *i* ∈ *I*, and

(15) $$\int f_{I}\;dp_{i}=0,\;\text{if}\;i\in I.$$

Using Eq. (15), it can be shown that, if *I, J* are two distinct subsets of *D*, then ∫ *f_I_f_J_ d***p** = 0. Hence, *f_I_* and *f_J_* are orthogonal if *I* ≠ *J*. In other words, Eqs. (14) and (15) show that a square-integrable function *f* can be decomposed into lower dimensional orthogonal functions. Sobol’ [30] also gives an analytical method to evaluate *f_I_* recursively. Furthermore, if we consider the parameters to be independent and uniformly distributed over their prescribed interval, Sobol’ showed that the variance in the distribution of the output, resulting from the variation in the parameters, can be decomposed as,

(16) $$V(Y)=\sum_{I\subset D}\int f_{I}^{2}dp,$$

where *Y* is the random variable corresponding to the output *y* = *f*(*p*_1_, …, *p_N_*) and ∫ · *d***p** represents integral over the parameters *p*_1_, …, *p_N_*. This gives an ANOVA-like decomposition of the variance of the output by partitioning it into contributions from uncertainties in the parameters, considering not only the effects of uncertainty in an individual parameter but also the uncertainty propagation due to functional dependence of the output on any interaction between the parameters. For example, $\int f_{\left\{1\right\}}^{2}dp$ will be the contribution of the uncertainty in the parameter *p*_1_ to the uncertainty in the output due to the functional dependence of the output on the parameter *p*_1_, and $\int f_{\left\{1,2\right\}}^{2}dp$ will be the contribution of the uncertainty in the parameters *p*_1_ and *p*_2_ to the uncertainty in the output due to the functional dependence of the output on the parameters *p*_1_ and *p*_2_ **as captured by *f*_{1,2}_**. In this case, the contribution of $\int f_{\left\{1\right\}}^{2}dp$ is referred to as the *main* effect or the *first-order* effect of the parameter *p*_1_, the contribution of $\int f_{\left\{1,2\right\}}^{2}dp$ is referred to as the *interaction* effect of the parameters *p*_1_ and *p*_2_.

The Sobol’ indices are then defined as,

(17) $$S_{I}=\frac{\int f_{I}^{2}\;dp}{V\left(Y\right)}.$$

Intuitively, the ranking of $S_{I}$ will induce a ranking of the contribution of the corresponding Sobol’ function *f_I_* to the variance of the output *Y*. We denote the random variables corresponding to the output *y* and a parameter *p_i_*, by *Y* and *P_i_*, respectively. Then, to consider the total contribution of the variance in *P_i_* to the variance of *Y*, contributions from all of the Sobol’ functions of *p_i_* should be considered. Following this line of reasoning, the *total* Sobol’ index for a parameter *P_i_*, is defined by,

(18) $${\bar{S}}_{i}=\frac{\underset{\begin{array}{l} I\subset D\\ i\in I \end{array}}{\Sigma }\int f_{I}^{2}\;dp}{V\left(Y\right)}.$$

It follows from Eq. (18) that, if ${\bar{S}}_{i}=0$, then *f_I_* = 0 for all *I* ⊂ *D* where *i* ⊂ *I*. This shows that the QoI does not functionally depend upon the input *p_i_* in the chosen parameter space, $\Omega =\prod_{i=1}^{N}[a_{i},b_{i}]$, and we can conclude that the presumed variation in the parameter *p_i_* is of no consequence to the QoI. Hence, in the sensitivity analysis using SIs, a parameter is deemed as *unimportant* if the corresponding total SI is negligible. Consequently, the choice of the value of the parameter *p_i_*, in the analyzed parameter space, will not have any effect on the QoI.

Furthermore, a statistical interpretation for the total Sobol’ index can be seen from

(19) $${\bar{S}}_{i}=\frac{E_{P_{∼i}}\left[V_{P_{i}}\left(Y|P_{∼i}\right)\right]}{V\left(Y\right)},$$

where **P**_~*i*_ is the vector of random variables for all parameters *except p_i_* [39]. *V_Pi_*(*Y*|**P**_~*i*_) is the conditional variance in the output if we vary only the parameter *p_i_* and fix all of the remaining parameters, giving a measure of local sensitivity of the QoI to *p_i_*. For a global sensitivity measure, variation in all of the parameters has to be considered simultaneously, and as a result, we can consider the statistical distribution of *V_Pi_*(*Y*|**P**_~*i*_) over the *N* — 1 dimensional space corresponding to **P**_~*i*_. ${\bar{S}}_{i}$ then measures the expected value of this distribution, i.e. the expected value of the conditional variation in the output with respect to *p_i_* over the space of all of the remaining parameters (*E*_**P**_~*i*__ [*V_P_i__*(*Y*|**P**_~*i*_)]), normalized by the variance in the output (*V*(*Y*)). A high ${\bar{S}}_{i}$ therefore indicates that we expect the variance in the output to be high with respect to the variance in *p_i_*, considering simultaneous variation in all of the remaining parameters as well, and we classify it as a *significant* parameter. On the other hand, a negligible ${\bar{S}}_{i}$ will indicate that we do not expect any variance in the output with respect to *p_i_* and it can be frozen at any value in the [*a_i_, b_i_*] without significantly affecting the QoI over the entire parameter space *Ω*.

The *first-order* or *main* Sobol’ indices are defined by,

(20) $${\underline{S}}_{i}=\frac{\int f_{\left\{i\right\}}^{2}\;dp}{V\left(Y\right)}.$$

We define the *additional* SI of a parameter *p_i_* as the contributions of all of the interaction effects involving the parameter *p_i_*, and denote it by $A_{i}$. Hence,

(21) $$A_{i}={\bar{S}}_{i}-{\underline{S}}_{i}\;=\frac{\underset{\begin{array}{l} I\subset D,i\in I\\ I\neq \{i\} \end{array}}{\Sigma }\int f_{I}^{2}\;dp}{V(Y)}$$

Further analysis reveals the following relations between main Sobol’ indices and statistics of the QoI-distribution [39],

(22) $${\underline{S}}_{i}=\frac{E_{P_{i}}\left[V\left(Y\right)-V_{P_{∼i}}\left(Y|P_{i}\right)\right]}{V\left(Y\right)},\text{ and}$$

(23) $${\underline{S}}_{i}=\frac{V_{P_{i}}\left(E_{P_{\sim i}}\left[Y|P_{i}\right]\right)}{V\left(Y\right)}.$$

It follows from Eq. (22), that high ${\underline{S}}_{i}$ indicates a high expectation of a reduction in the variance of the QoI if the value of *P_i_* is known.

### 3.2 Numerical estimation of SIs

Since the integrals required to define SIs might not always be determinable analytically, numerical methods are often used to estimate these indices. The available methods can be classified into three categories: hybrid pick-and-freeze, extended Fourier amplitude sensitivity test (eFAST), and generalized polynomial chaos expansion (gPC). The concept for hybrid pick-and-freeze was provided by Sobol’ in his seminal paper for SIs [30], in which he used Monte-Carlo methods to estimate the integrals corresponding to the SIs. The Monte-Carlo scheme has since been improved, and reviews and comparisons of different hybrid pick-and-freeze strategies can be found in [39, 41].

In this work, we implement the hybrid pick-and-freeze of Jansen ([42]) to estimate total SIs. Jansen’s scheme has been shown to be more efficient than similar sampling-based schemes, especially when used in conjunction with quasi-random sequences [39, 41]. To estimate the first-order SI, we implemented the “Correlation 2” scheme proposed in [41]. This scheme uses a larger number of samples than many other first-order SI estimators, but we prefer this scheme because it has been shown to provide a better estimation in the case when the SIs for some of the parameters may be small. For more information on the estimation of Sobol’ indices, see Appendix D.

## 4 Results

As previously stated, our Quantity of Interest will be the steady-state pH at the left boundary (epithelial wall)

$${\text{QoI = -log}}_{\text{10}}(C_{\text{H}}{|}_{x=0}).$$

Intuitively, a large SI for a given parameter means that fluctuations in that quantity may cause large fluctuations in the pH experienced by the mucosal tissue of the stomach and potentially be detrimental to stomach health. Conversely, a small SI indicates that variations in that parameter do not appreciably contribute to variations in the pH at the stomach wall, which could be physiologically advantageous.

### 4.1 Full Sobol’ Analysis

One of the central results from [18] was the quantification of the mucosal pH as a function of *k*_HI_ and *k*_AB_. In that study, all other parameters were fixed using values estimated from biological data. It was shown that the mucosal pH only varied appreciably in the regime of low *k*_HI_ and/or low *k*_AB_. Here, we extend that study by including simultaneous variation in other model parameters (due to different experimental conditions, or simply natural variations between people) and use Sobol’ indices for sensitivity analysis. Table 1 shows all of the parameters of interest, their physical units, and the intervals on which we sample them. The final column also indicates which parameters were assumed to be log-uniformly distributed (as opposed to uniformly distributed).

**Fig. 2:**
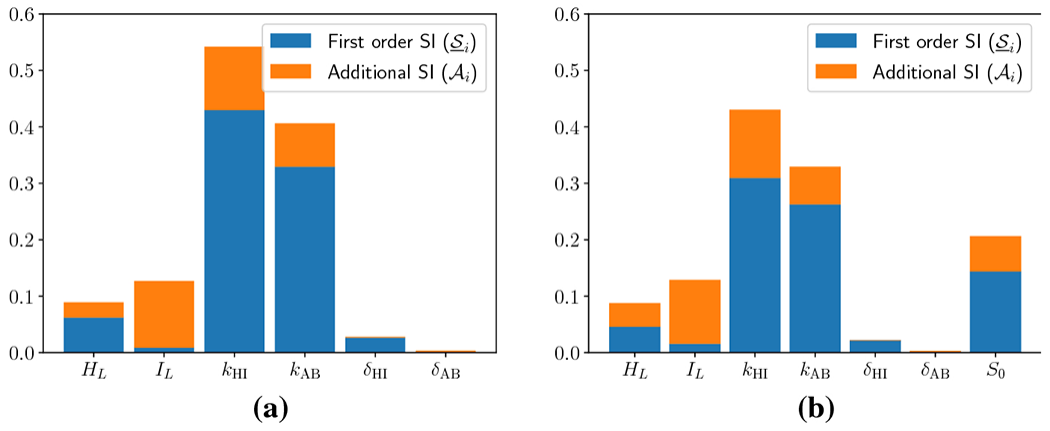
Sobol’ indices for boundary pH for each parameter. First order (${\underline{S}}_{i}$) and additional ($A_{I}$) SI are depicted, while their sum indicates total SI (${\bar{S}}_{i}$). (a) Source is fixed at 1, and all other parameters are varied in their region of interest, (b) All seven parameters are varied.

We begin with varying all of the parameters except for *S*_0_, which is fixed at its nominal value of 1. This set of parameters captures those that were either varied, or roughly estimated in [18], and thus represents an approximate reproduction of the analysis from that work using the SI methodology. The estimated SIs are shown in Fig. 2a. The blue bars indicate estimated first-order SI (${\underline{S}}_{i}$), while the orange bars indicate the additional SI ($A_{i}$). The combined height of the orange and blue bars gives the total SI for each variable (${\bar{S}}_{i}$). It is immediately clear that the two parameters with the largest total SI are the ion exchange rates (*k*_HI_ and *k*_AB_ and the bias constants *δ*_HI_ and *δ*_AB_) have negligible total and first-order SIs. Furthermore, any variations in the lumenal concentrations (*H*_L_ and *I*_L_ account for strictly less than 20% of the variance in the QoI in this parameter regime, with their combined first-order effects accounting for less than 7% of the variance in the QoI. Therefore, the Sobol’ analysis shows that variations in *k*_HI_ and *k*_AB_ are necessary and sufficient to bring about a significant variation in the QoI, without any significant effects of variations in the other parameters. In this regard, Sobol’ analysis supports and extends the work in [18]. Together with the previous study, our analysis implies a remarkably robust mechanism for gastric pH maintenance.

Next, we also include the effects of variation in the magnitude of the Hydrogen secretion, *S*_0_. The estimated SIs are shown Fig. 2b. Based on the convergence criterion that we define in Appendix D, 20000 evaluations of the QoI were required to estimate the total SIs and 48000 evaluations were required to estimate the first-order SIs. The mean of the evaluations used to estimate total SI is around 6.235 with a variance of 0.789. The comparison between total SIs of parameters again shows that the two rate constants are the most significant. The remaining parameters fall roughly into three categories. The bias constants *δ*_HI_ and *δ*_AB_ still have negligible SI (< 0.05). The lumenal boundary concentrations *H_L_* and *I_L_* have small SI (~ 0.1). However, the ion source magnitude *S*_0_ has significant SI (~ 0.2). This means that variation in *S*_0_ cannot be entirely ignored since it has non-negligible total SI along with a significant first-order effect – varying *S*_0_ by itself can account for 15% of the variance in the QoI.

**Fig. 3:**
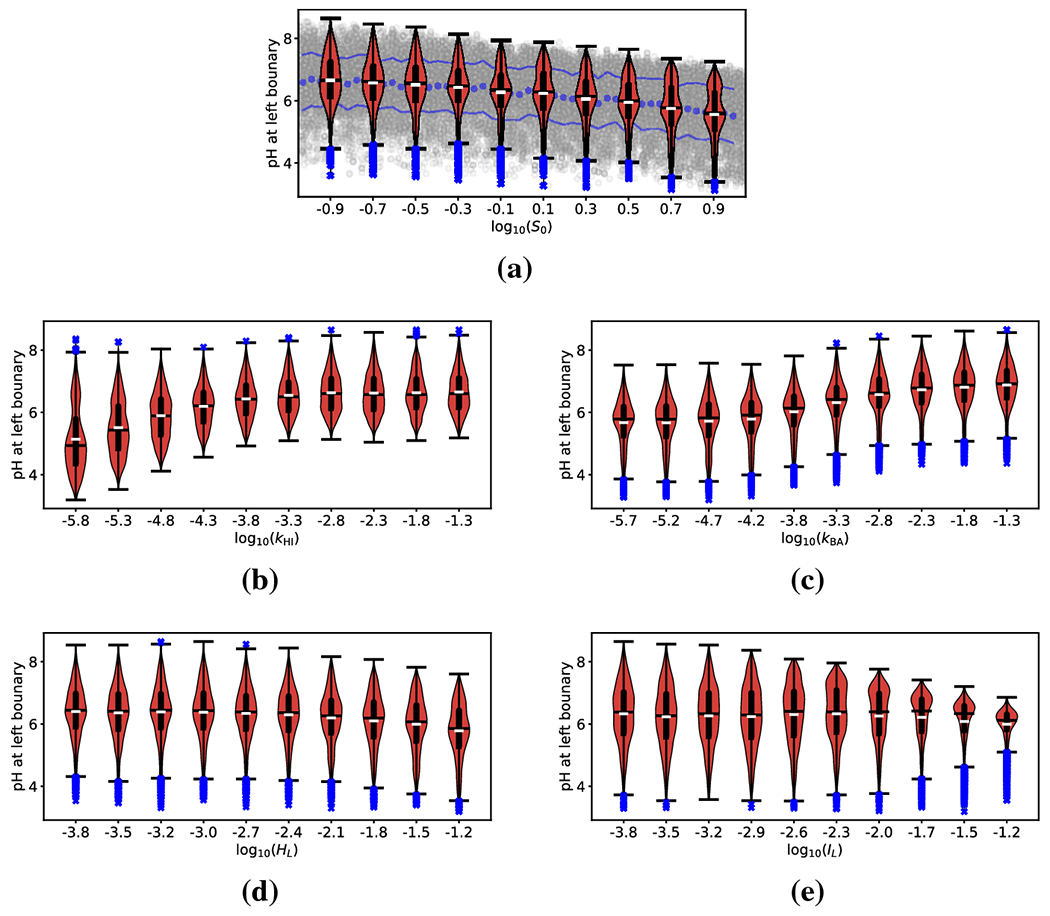
Violin and box plots for log_10_ (*H*_0_ as a function of (a) ion source magnitude (*S*_0_), (b) hydrogen exchange rate (*k*_HI_), (c) bicarbonate exchange rate (*k*_AB_), (d) lumenal hydrogen concentration (*H*_L_), and (e) lumenal cation concentration (*I*_L_). White hashes indicate mean of QoI within a subinterval and black hashes indicate the median. Thick black lines indicate the range from first to third quartiles. Black whiskers indicate the extent of the data (sans outliers), and blue x-es indicate individual outliers (median ±1.5 times inter-quartile range). Panel (a) also shows the underlying data (with running mean and mean±std. indicated) for illustrative purposes.

To more precisely describe the impact each parameter has on the model predictions, we use violin/box-and-whisker plots of the QoI as each parameter is varied (see Fig. 3). Figure 3a illustrates the process of generating these plots for the parameter *S*_0_. The grey points indicate a simple scatter of the measured QoI as a function of the parameter *S*_0_. The dashed and solid purple lines indicate a running mean and mean ± standard deviation of the QoI. However, this format makes it difficult to visually infer trends in the distribution of the QoI as *S*_0_ is varied. Therefore, we divide the range of *S*_0_ into ten equal width sub-intervals and generate a violin for each. The white hash indicates the mean of the QoI for samples in that respective subinterval and the black hash indicates the median. The thicker black line indicates the region between the first and third quartiles. The black whiskers indicate the extent of the data (sans outliers), and the blue x-es indicate individual outliers defined as median ±1.5 times the inter-quartile range. Finally, the width of the red violins represent a smoothed histogram of the data within each subinterval. For visual clarity we present only the violin plots, and not the underlying scatter plots, for all remaining parameters.

Visual inspection of Fig. 3a shows that the mean value of wall pH changes by approximately 1 as *S*_0_ is varied over the given interval. From Eq. (23), we can interpret the ratio of this variation in the expected value of QoI to the total variation of QoI as the the first-order SI of the parameter under consideration (${\underline{S}}_{S_{0}}$). Other changes in the distribution of the QoI (from violin to violin) are attributable to additional SI of that parameter ($A_{S_{0}}$). For example, the distribution of QoI is bimodal when *S*_0_ is large but unimodal when *S*_0_ is small.

The observed change in mean QoI also has a very natural and unsurprising physiological interpretation: when the source of hydrogen due to gastric secretion is larger, the gastric wall (on average) is more acidic. However, we note here that variations in secretion rate (*S*_0_) over two orders of magnitude produces a variation in mean gastric pH of less than one. Thus, even though *S*_0_ exhibits a significant total SI, the mean mucosal pH is well within biologically acceptable limits (≈ 6 or 7). This seems physiologically advantageous, as one would expect *in-vivo* gastric secretion to vary throughout the day in response to various nervous and endocrine stimuli. In Section 4.2, we investigate more closely the behavior of mucosal pH in the limit of large *S*_0_ (i.e. secretion in response to a recent meal or histamine stimulus) and small *S*_0_ (i.e. secretion in response to weak/no stimulus).

Figures 3b and 3c show violin plots for QoI as a function of *k*_HI_ and *k*_AB_. As expected, because these two parameters have the largest first order SI, we observe a notable variation in the mean of the QoI. As either exchange rate parameter is decreased, the measured pH decreases (on average), implying acidification of the gastric mucsoal wall and suggesting a failure to maintain homeostasis. This is in line with the results presented in [18], which indicated that as long as both exchange rates were sufficiently large (i.e. sufficiently rapid exchange of ions between the epithelium and gastric lumen), a healthy pH was robustly maintained. The present analysis also allows us to determine how the variance in the predicted wall pH changes as a function of ion exchange rates. In particular, we can see that when *k*_HI_ is low, there is a significant increase in the variation of the QoI. As this behavior affects higher order moments of the QoI distribution, it may be related to the additional affects within the total SI of *k*_HI_. A similar (but distinct) result was presented in [18], where it was shown that mucosal pH could vary wildly when *k*_HI_ = 0. In Section 4.3 we investigate more closely the behavior of mucosal pH in the limit of small *k*_HI_. Finally, we note that the parameter *k*_AB_ does not obviously affect the spread of the distribution of QoI, and therefore exhibits less additional SI ($A_{k_{\text{AB}}}<A_{k_{\text{HI}}}$).

The parameters *H_L_* and *I_L_* both have relatively small SIs. These parameters represent the concentration of ions in the interior of the stomach lumen, which could vary in the *in vivo* system of interest (in response to diet, for example). Small SI imply that the pH experienced by the stomach wall does not vary appreciably in response to these temporal fluctuations, implying a robust physiological control mechanism. In this regard, the data presented here represents a more quantitative (and global) generalization of the argument put forward in [18]. We note however, that these SIs are not completely negligible and are comprised of significant additional SI. For this reason we include the violin plots of both in Figs. 3d and 3e. Of particular note is that even though the mean QoI does not depend strongly on *I_L_* (${\underline{S}}_{I_{L}}$ is small), when *I_L_* is large the variation in QoI is drastically decreased. In this regard, one could describe the model as extremely *robust* in the limit that *I_L_* is large: regardless of other parameter values, the pH at the stomach wall will almost certainly be ≈ 6.

We do not include violin plots of *δ*_HI_ and *δ*_AB_ in this section, as both of those parameters have negligible SI. However, those graphs can be found in Appendix A for completeness. We also note here that there is no “accepted” value for these parameters in the literature. In [18] their values were roughly estimated based on data available from other epithelial cell types. The negligible SI for *δ*_HI_ and *δ*_AB_ imply that the results presented in [18] do not depend on the these estimated values and are generically representative of the model behavior.

### 4.2 Analysis for Fixed Source Magnitude

The results of Section 4.1 indicate that the ion exchange rates *k*_HI_ and *k*_AB_ exhibit the largest SI, which when interpreted in conjuction with [18], imply a robust physiological control of mucosal pH. However, they also indicate that the hydrogen source magnitude (or secretion rate) *S*_0_ may make a significant contribution to the sensitivity of the model predictions, which was not addressed in previous investigations. This is physiologically relevant, as the secretion rate of hydrogen in the human stomach is known to vary by a factor of 40 or more, in response to histamine levels or the recency of a meal [14, 22]. We analyze the sensitivity of the model predictions when secretion is at its extremal values, i.e. when the stomach is quiescent and not secreting much gastric juice, or after intense stimulation. In this section, we perform a second analysis where we fix the value of *S*_0_ and calculate the SI (primary and additional) for the remaining six parameters. This is done twice: with *S*_0_ = 0.1 and with *S*_0_ = 10.

#### Large *S*_0_:

**Fig. 4:**
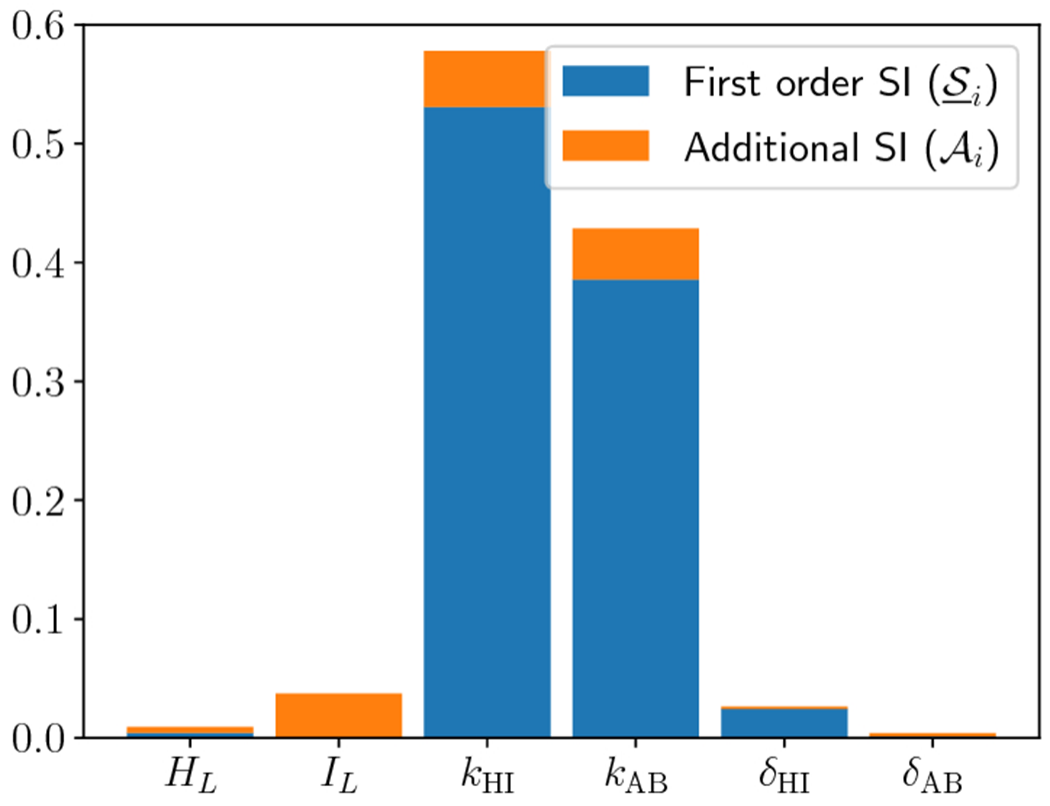
Sobol’ indices for the other six parameters when hydrogen source is fixed and large (*S*_0_ = 10). First order (${\underline{S}}_{i}$) and additional ($A_{I}$) SI are depicted, while their sum indicates total SI (${\bar{S}}_{i}$).

Upon fixing *S*_0_ = 10 (which represents stimulated gastric secretion), we recalculate the SI of the other six parameters. This requires approximately 18000 evaluations of the QoI. The primary and additional SI are shown in Fig. 4. In this regime, *k*_HI_ and *k*_AB_ remain the dominant SI, and are predominantly characterized by first order SI. Remarkably, no other parameters exhibit significant SI. This can be interpreted to mean that in the regime of strong gastric secretion, the ion exchange rates nearly completely govern the mucosal pH.

**Fig. 5:**
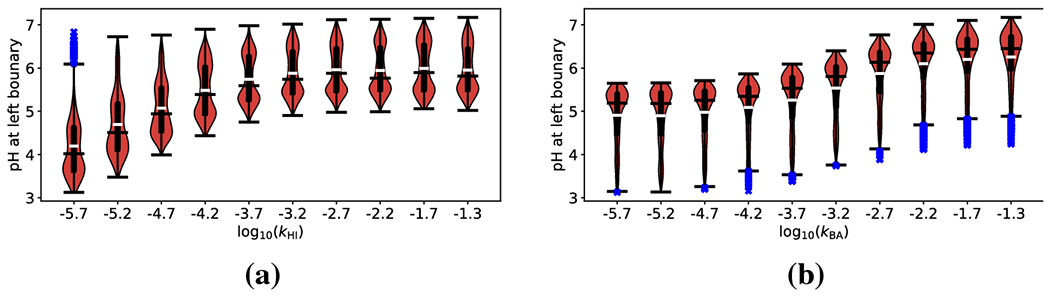
Violin and box plots for log_10_ (*H*_0_) as a function of (a) hydrogen exchange rate (*k*_HI_) and (b) bicarbonate exchange rate (*k*_AB_), when hydrogen source is large (*S*_0_ = 10). White hashes indicate mean of Qol within a subinterval and black hashes indicate the median. Thick black lines indicate the range from first to third quartiles. Black whiskers indicate the extent of the data (sans outliers), and blue x-es indicate individual outliers (median ±1.5 times inter-quartile range).

Figure 5 shows the violin plots for QoI as a function of *k*_HI_ and *k*_AB_. Consistent with the SI shown in Fig. 4, when ion exchange rates are small the gastric wall is likely to be significantly more acidic. We note that there is a marked increase in the variation of QoI when *k*_HI_ is small and this is the only parameter regime when the wall pH is strongly acidic (< 4). This result is also consistent with those presented in [18]: as long as ion exchange rates are “fast enough”, the mucosal wall is generically maintained at near-neutral pH. The violin plots for the remaining four parameters do not add much to our discussion here (and their SI are insignificant), but we include them in Appendix A for completeness.

#### Small *S*_0_:

**Fig. 6:**
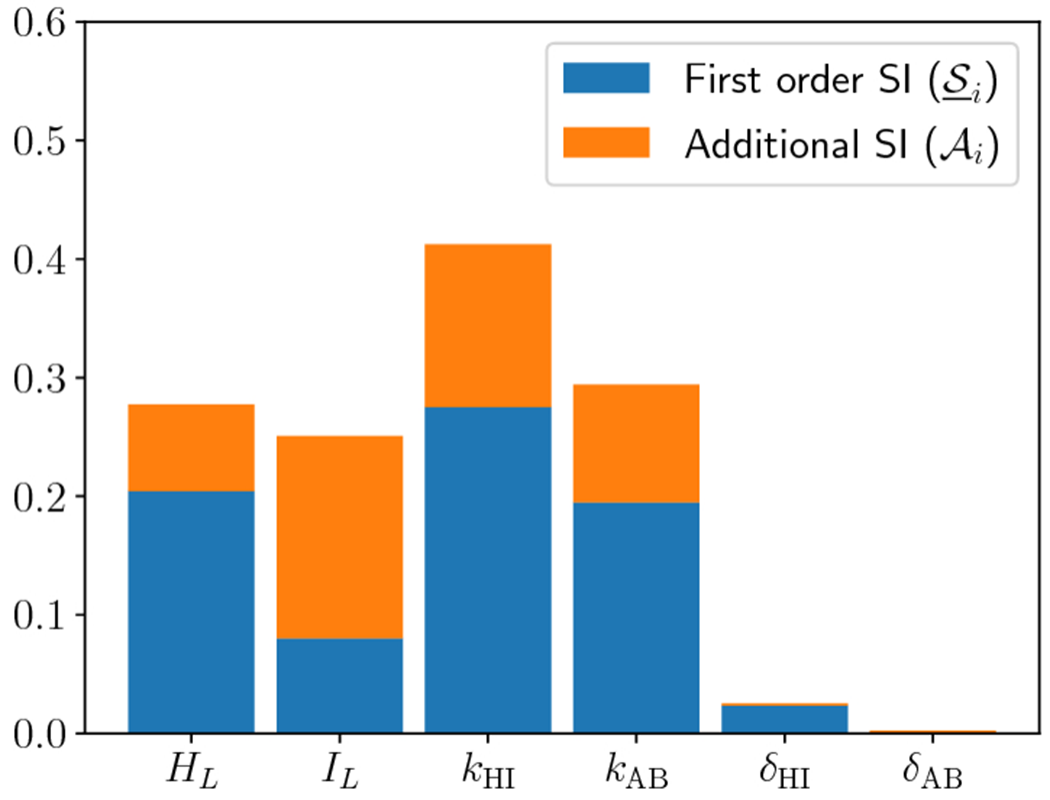
Sobol’ indices for the other six parameters when hydrogen source is fixed and small (*S*_0_ = 0.1). First order (${\underline{S}}_{i}$) and additional ($A_{I}$) SI are depicted, while their sum indicates total SI (${\bar{S}}_{i}$).

After fixing *S*_0_ = 0.1 (which represents suppressed secretion of gastric juice), we again calculate the SI of the remaining parameters. Convergence required roughly 20000 evaluations of the QoI, and the calculated SI are shown in Fig. 6. The ion exchange rates still exhibit the largest SI; however in this regime we also observe significant SI for the lumenal ion concentrations *H_L_* and *I_L_*. This implies that the mucosal pH is more sensitive to the ionic composition of the gastric interior when secretion is suppressed. Furthermore, all four parameters exhibit appreciable additional SI, implying that there are significant interaction effects between parameters. As in all other analyses, the SI of both offset parameters is negligible.

**Fig. 7:**
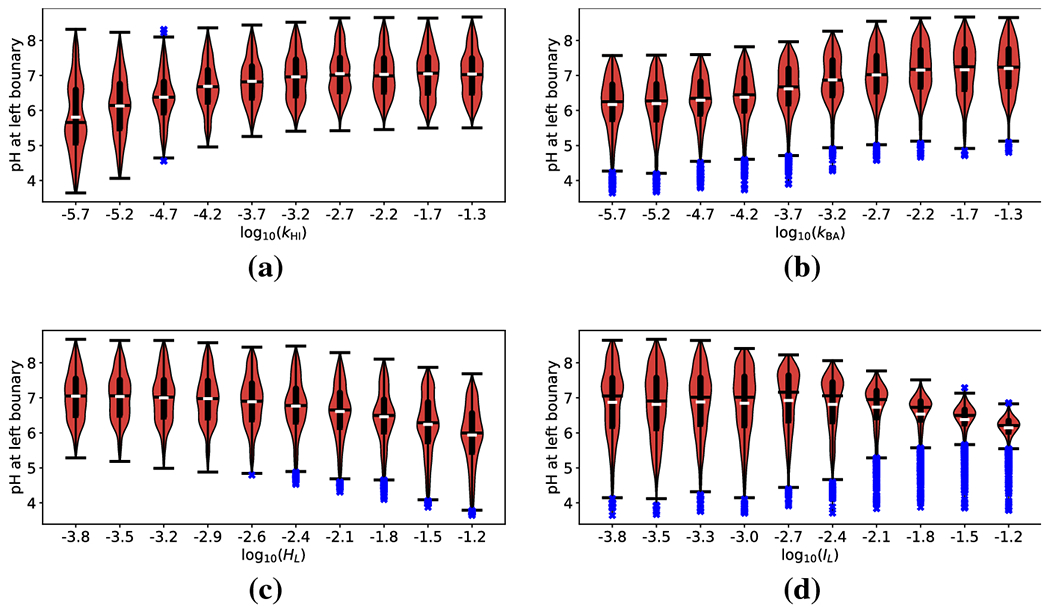
Violin and box plots for log_10_ (*H*_0_) as a function of (a) hydrogen exchange rate (*k*_HI_), (b) bicarbonate exchange rate (*k*_AB_, (c) lumenal hydrogen concentration (*H_L_*), and (d) lumenal cation concentration (*I_L_*), when hydrogen source is small (*S*_0_ = 0.1). White hashes indicate mean of QoI within a subinterval and black hashes indicate the median. Thick black lines indicate the range from first to third quartiles. Black whiskers indicate the extent of the data (sans outliers), and blue x-es indicate individual outliers (median ±1.5 times inter-quartile range).

Figures 7a and 7b show the violin plots for *k*_HI_ and *k*_AB_. As in all previous analyses, we observe that increased ion exchange generically leads to a less acidic mucosal wall. However, the mean QoI exhibits a relatively weak dependence on either exchange rate parameter. This is consistent with the relatively small first order SI shown in Fig. 6. We also note here that the mucosal wall is generically neutral (or near neutral), always with a mean pH ≳ 5.5. This is physiologically unsurprising, as these results are produced in the regime where less hydrogen is being secreted into the gastric lumen.

Figures 7c and 7d show the violin plots for *H_L_* and *I_L_*. Figure 7c shows that an increase in *H_L_* (which represents a more acidic gastric lumen) likely leads to a more acidic mucosal wall. However, it is also associated with an increase in the variance of the QoI. This means that when secretion is low, the mucosal pH is more susceptible to variations in lumenal acidity but may be able to maintain neutral (or near neutral) conditions depending on other factors. Conversely, Fig. 7d shows that the QoI exhibits a drastic decrease in variance associated with high values of *I_L_*. This effect is even more pronounced than that observed in Section 4.1. The analysis shows that when gastric secretion is low, but sodium concentration in the stomach lumen is high, the mucosal pH will *nearly* always be ≈ 6.

### 4.3 Sobol’ Analysis for low *k*_HI_

A recurring theme in the above sections is that *k*_HI_ exhibits a greater SI than the other parameters considered. Physically, this is somewhat surprising, as the analysis in [18] showed that under most conditions, the flux of hydrogen ions through hydrogen/sodium exchange is significantly smaller than the other sources/sinks of hydrogen in the system (secretion and bicarbonate buffering). However, the analysis of [18] also concluded that *k*_HI_ is a critical parameter of the system: when it is identically zero (representing a lack of hydrogen/sodium exchange proteins), the system fails to robustly maintain a near-neutral mucosal pH. Our analysis here has corroborated this finding; in all cases we see that low values of *k*_HI_ lead to drastically increased variance in QoI.

To further explore the behavior of the system in the limit of “vanishing” hydrogen/sodium exchange, we perform a series of calculations where *k*_HI_ is fixed at a low values, and the SI of the remaining six parameter are calculated. We calculate the SI of the remaining parameters for five values of *k*_HI_ ranging from 10^−6^ to 10^−8^. Calculating these SI required roughly 24000 to 26000 evaluations of the QoI, depending on the value of *k*_HI_. We do not explore the case when *k*_HI_ = 0, as this caused the linear operators associated with our numerical scheme (see Appendices B and C) to become ill-conditioned.

**Fig. 8:**
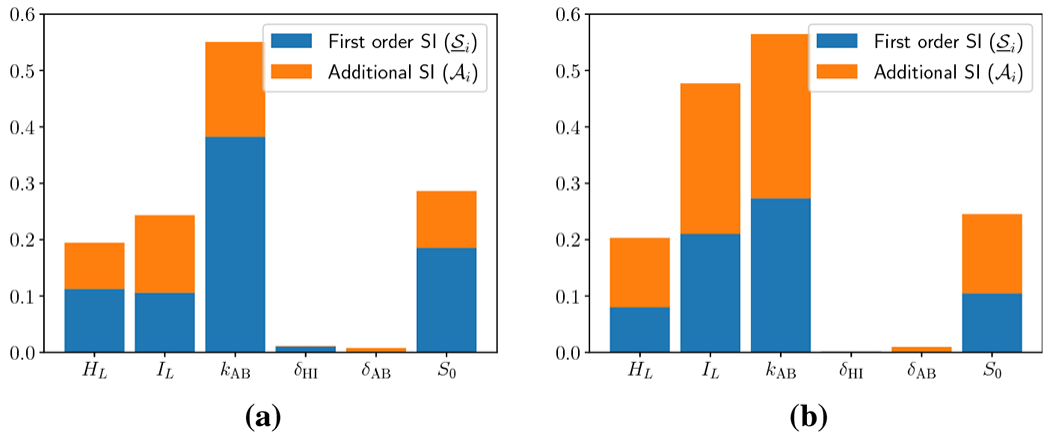
First order and additional Sobol’ indices for the other six parameters when hydrogen exchange rate is fixed and small. Panels show (a) *k*_HI_ = 10^−6^ and (b) *k*_HI_ = 10^−8^. First order (${\underline{S}}_{i}$) and additional ($A_{I}$) SI are depicted, while their sum indicates total SI (${\bar{S}}_{i}$).

Figure 8a shows the SI of the remaining six parameters when *k*_HI_ = 10^−6^; Fig. 8b shows the same SI when *k*_HI_ = 10^−8^. In both Figs. 8a and 8b, we see that though the numerical values have changed, the SI of the remaining parameters are qualitatively similar to the full Sobol’ analysis: *k*_AB_ exhibits the dominant SI, followed by *S*_0_, *I_L_*, and *H_L_* with moderate SI, and finally the ion exchange offsets (*δ*_HI_ and *δ*_AB_) have negligible SI.

**Fig. 9:**
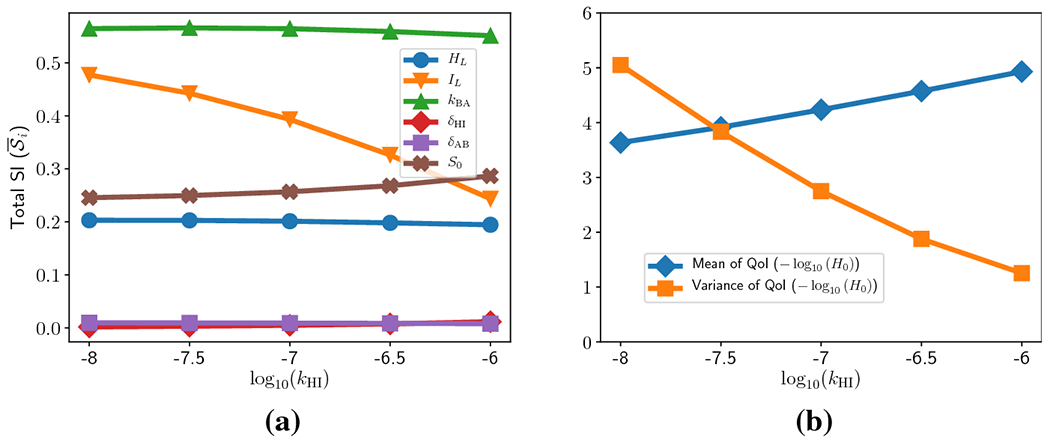
(a) Total Sobol’ indices of the other six parameters as a function of *k*_HI_. (b) Mean (blue diamonds) and variance (orange squares) of pH at the left boundary as a function of *k*_HI_.

Comparing Fig. 8a to Fig. 8b, we observe two trends. First, we note that decreasing *k*_HI_ leads to significant increase in total SI for *I_L_* and a moderate increase in the SI of *H_L_*. This can be interpreted to mean that when there is a deficiency in (or shortage of) hydrogen/sodium exchange proteins in the epithelial wall, mucosal pH becomes much more sensitive to the ionic composition of the stomach lumen. To more clearly illustrate this phenomenon, Fig. 9a shows the total SI for the remaining size parameters as a function of *k*_HI_. It clearly illustrates the increase of ${\bar{S}}_{I_{L}}$ as *k*_HI_ → 0. One can also observe moderate increases in ${\bar{S}}_{H_{L}}$ and ${\bar{S}}_{k_{\text{AB}}}$, as well as a moderate decrease in ${\bar{S}}_{S_{0}}$. Generically, the SI for ion exchange offset parameters is negligible. These analyses all corroborate and extend the work presented in [18].

The second trend of note in Fig. 8 is that decreasing *k*_HI_ appears to decrease the first order SI and increase the additional SI for all parameters. This indicates that are significant interaction effects and may suggest variation in higher order moments of the QoI as parameter values change. For this reason we plot the mean and variance (over all evaluations used to calculate the SI) of the mucosal pH as a function of *k*_HI_. The results are shown in Fig. 9b. As can be seen, the mucosal pH becomes (on average) more acidic as *k*_HI_ decreases. However, there is also a dramatic (> 3-fold) increase in the variance of mucosal pH; the coefficient of variation for the mucosal pH increases from approximately 0.23 (when *k*_HI_ = 10^−6^) to approximately 0.62 (when *k*_HI_ = 10^−8^). Taken together, these facts imply that as hydrogen/sodium exchange is down-regulated, the bicarbonate buffering mechanism becomes unable to robustly maintain a neutral environment adjacent to the mucosal wall.

## 5 Discussion

In this paper, we have presented a global sensitivity analysis of steady state epithelial pH predicted by an electrodiffusive model of hydrogen transport in the gastric mucus layer. Using Sobol’ Indices, we have provided a systematic extension of the (relatively local) sensitivity analysis of this model reported in previous work. Our results support the existing analysis of [18], while also providing further physiological insight by considering variation in biological parameters which were considered “fixed” in previous works.

The analysis presented here repeatedly shows that the rate constants of ion exchange through the epithelial surface (*k*_HI_ and *k*_AB_) are the dominant parameters that impact the maintenance of the pH at the epithelial wall. These two parameters exhibited the largest sensitivities in all regimes considered, particularly when the source of hydrogen secretion is large (i.e. stimulated gastric secretion). In the regime of suppressed gastric acid secretion, the parameters representing the concentration of ions in the gastric lumen exhibited significant sensitivities. Taken together, these results indicate that when the stomach is not stimulated (and not producing large amounts of gastric juice), the contents of the stomach may impact the pH of the epithelium. However, when hydrochloric acid is actively introduced to the stomach lumen (i.e. secretion is stimulated by ingesting a meal, or treatment with histamine) the exchange of ions at the epithelial wall becomes the dominant process and exerts significant control over wall pH.

Furthermore, the large sensitivities of *both* exchange rate parameters (*k*_HI_ and *k*_AB_) suggest that both hydrogen/sodium *and* bicarbonate/chloride exchange events are critical to the control of the hydrogen concentration adjacent to the gastric epithelium. This conclusion is in line with the work of [18], but to our knowledge has not been suggested anywhere in the gastric physiology literature, where the physiological purpose of the hydrogen/sodium exchange proteins is rarely, if ever, discussed.

The observation that epithelial pH is sensitive to hydrogen/sodium exchange even though this relationship is not appreciated in the physiology literature, together with the analysis of [18], inspired us to perform a sequence of sensitivity analyses where the SI of the remaining parameters were calculated for several (small and decreasing) values of *k*_HI_. Notably, the sensitivity measure of lumenal sodium concentration increases. This is perhaps unsurprising, as the electroneutrality constraint implies that the concentration of sodium and hydrogen at the epithelial wall are strongly dependent on one another, and impeding the regulatory function of hydrogen/sodium exchange at the epithelium would reasonably leave epithelial pH more sensitive to sodium diffusing from the lumen. Perhaps more surprising is that the sensitivity measure of hydrogen source magnitude *decreases* as *k*_HI_ decreases. As the rate of hydrogen/sodium exchange decreases, one would expect the epithelium to become *more* sensitive to the amount of hydrogen secreted by the stomach. This illustrates a common misunderstanding in how SI are often interpreted in the broader community. Directly comparing SI values generated with two different parameter sets (*k*_HI_ = 10^−6^ cm/sec vs. *k*_HI_ = 10^−6^ cm/sec) may be misleading, as these values are expressed *as a proportion of the total variation in the QoI*, which may be changing (see below). What can be concluded is that as *k*_HI_ decreases, the QoI becomes *relatively* more sensitive to *I_L_* than *S*_0_, as ${\bar{S}}_{I_{L}}<{\bar{S}}_{S_{0}}$ when *k*_HI_ 10^−6^, but ${\bar{S}}_{I_{L}}>{\bar{S}}_{S_{0}}$ when *k*_HI_ = 10^−8^.

Finally and perhaps most importantly, our analysis shows that as the rate of hydrogen/sodium exchange decreases, the mean pH of the gastric epithelium also decreases, while the variance of this same quantity increases nearly five-fold. Both of these changes have drastic implications for healthy physiological function. A decrease in pH is generally detrimental to the health of the cells that make up the epithelial surface and is associated with numerous pathologies. Indeed, it is generally accepted that a major role of the gastric mucus layer is to protect the epithelium from the low pH of the lumen [11]. Therefore, any scenario which leads to a decrease in the lumenal pH would be physiologicaly disadvantageous. Furthermore, the increase in the variance as *k*_HI_ decreases implies that the epithelial pH is generally more sensitive to perturbations in the other parameters, and thus one could expect larger variations in wall pH. Taken together, these results imply something of a “double whammy” for the maintenance of healthy gastric pH: as the rate of hydrogen/sodium exchange decreases towards zero, the system is not only more likely to maintain an “unhealthy” pH at the wall, but it is more likely to experience large swings in said pH.

In closing, we would like to note that several of these results represent a powerful use for Sobol’ Indices (and sensitivity analysis in general) which remains underappreciated in the literature. Sensitivity analysis is often used to simply quantify uncertainty in model predictions associated with uncertainty in parameter estimation, and no deeper implications are considered. However, there are specific biological insights that can be determined by judicious use of sensitivity analysis – this includes varying the QoI, parameter regimes, and exploring the QoI distributions. Furthermore, the physical and/or biological quantities that model parameters represent are often not fixed, but rather dynamic quantities subject to random or deterministic fluctuations. Sensitivity analysis can be used to make quantitative inferences about the robustness of the system of interest to these fluctuations, and such inferences can have profound implications for how biological systems function in the face of a dynamic environment.

## Figures and Tables

**Table 1:** This table lists the relevant parameters for our Sobol’ analysis, their units, and the intervals over which we allow them to range. The final column indicates if the parameter is assumed to be log-uniformly distributed (otherwise, parameters are assumed to be uniformly distributed).

Parameter	Description	Units	Interval	Log-unif.

*k*_HI_	Rate constant of Hydrogen/Sodium Exchange	cm/sec	[1 × 10^−6^, 1 × 10^−1^]	**✓**
*k*_AB_	Rate constant of Chloride/Bicarbonate Exchange	cm/sec	[1 × 10^−6^, 1 × 10^−1^]	**✓**
*δ*_HI_	Bias constant of Hydrogen/Sodium Exchange	[−]	[5 × 10^4^, 15 × 10^4^]	
*δ*_AB_	Bias constant of Chloride/Bicarbonate Exchange	[−]	[5, 15]	
*H_L_*	Lumenal concentration of Hydrogen	M	[1 × 10^−4^, 1 × 10^−1^]	**✓**
*I_L_*	Lumenal concentration of cations	M	[1 × 10^−4^, 1 × 10^−1^]	**✓**
*S*_0_	Magnitude of Hydrogen secretion	[−]	[1 × 10^−1^, 1 × 10^1^]	**✓**

**Fig. A.1:**
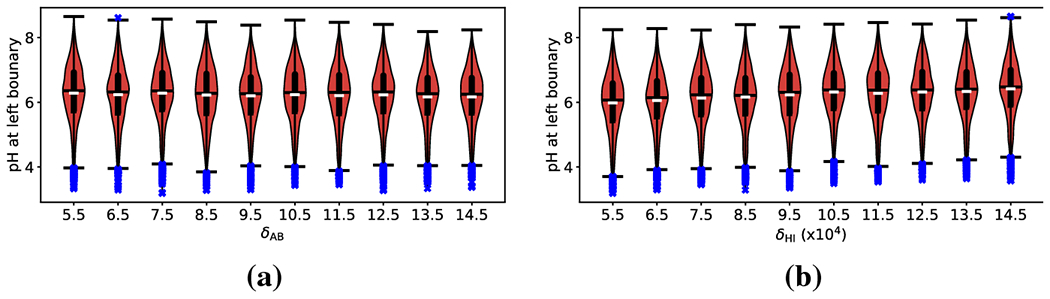
Violin and box plots for log_10_ (*H*_0_) as a function of (a) bicarbonate offset parameter *δ*_AB_, and (b) hydrogen offset parameter *δ*_HI_ during Sobol’ analysis of all 7 parameters.

**Fig. A.2:**
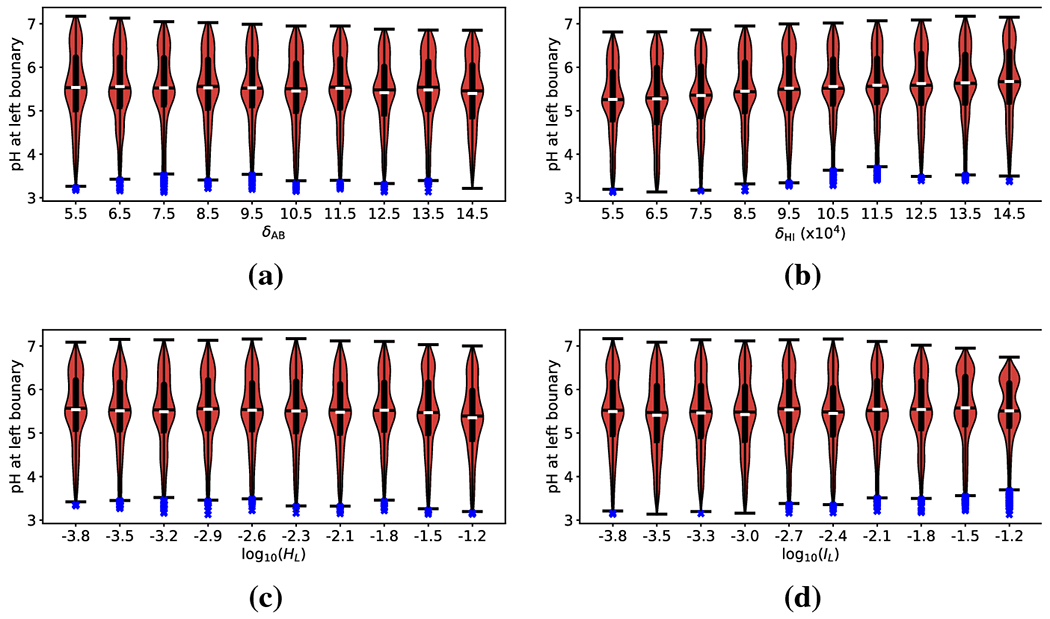
Violin and box plots for log_10_ (*H*_0_) as a function of (a) bicarbonate offset parameter (*δ*_AB_), (b) hydrogen offset parameter (*δ*_HI_), (c) lumenal hydrogen concentration *H_L_*, and (d) lumenal cation concentration *I_L_* when hydrogen source is large (*S*_0_ = 10).

**Fig. A.3:**
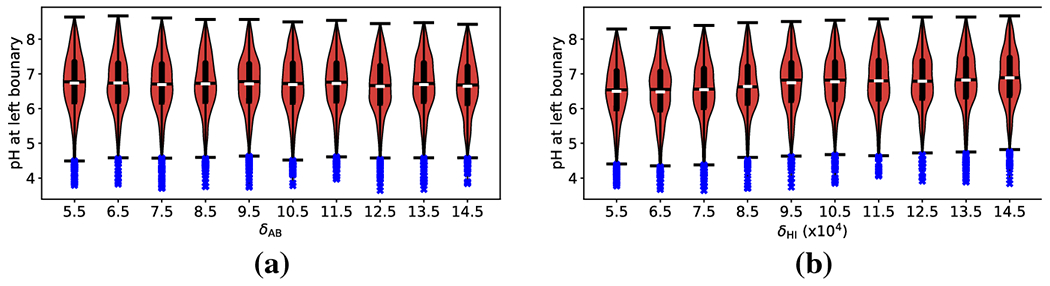
Violin and box plots for log_10_ (*H*_0_) as a function of (a) bicarbonate offset parameter (*δ*_AB_), and (b) hydrogen offset parameter *δ*_HI_, when hydrogen source is small (*S*_0_ = 0.1).

**Fig. C.4:**
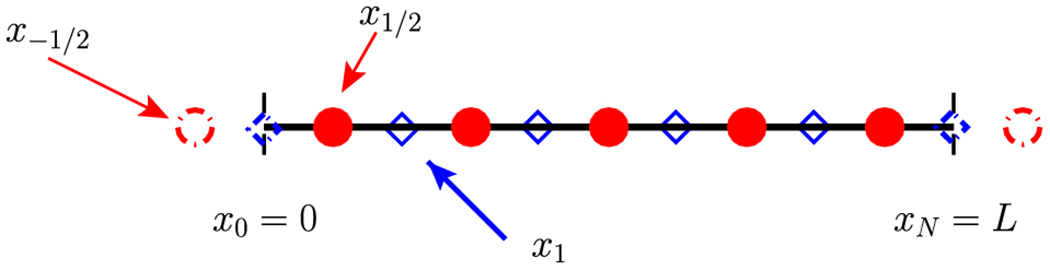
A schematic representation of our computational grid for *N* = 5. Dashed vertical black lines indicate the boundaries of the computational domain. Circles indicate cell centers, while diamonds indicate cell edges. Interior points are drawn with solid lines while ghost points are drawn with dash-dot lines.

**Fig. D.5:**
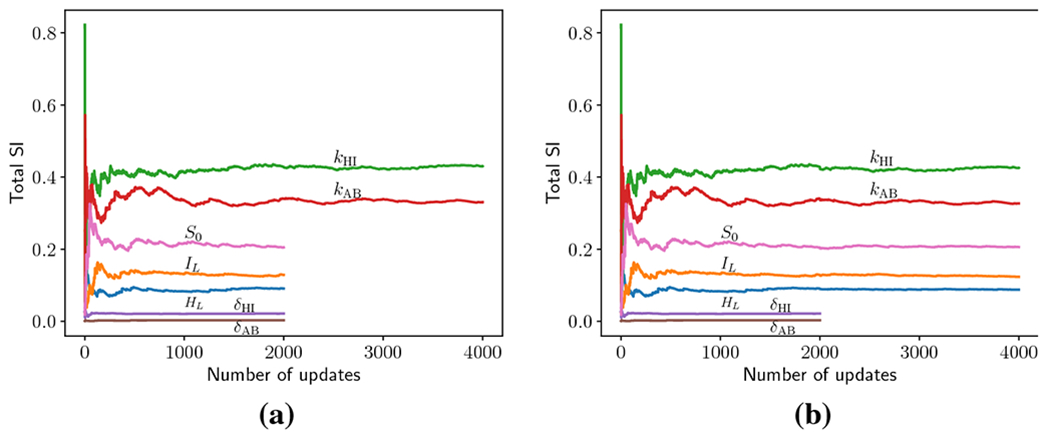
Convergence plots for total SI estimates, when all of the parameters are considered when (a) tol = 0.02 and (b) tol = 0.01, both with batch size *k* = 2000. At the lower tol value, some of the parameters take a greater number of updates for convergence. Note that the number of updates required for convergence for a parameter will always be a multiple of the batch size *k*.

## Appendices

### A Extra Figures

Here we present the remaining violin plots from the SI calculations in Sections 4.1 and 4.2. These parameters all exhibit small SI, and were therefore deemed “unimportant” for discussion We include these figures for completeness.

### B Iterative Solution Scheme

To determine the steady state ion concentrations (and then evaluate the QoI), we must solve the following system of five equations

(B.1) $$\frac{\partial }{\partial x}\left(u\left(x\right)C_{\text{H}}\right)=\frac{1}{\theta_{s}\left(x\right)}\frac{\partial }{\partial x}\left(\theta_{s}\left(x\right)\left(D_{\text{H}}\frac{\partial }{\partial x}C_{\text{H}}+z_{\text{H}}D_{\text{H}}C_{\text{H}}\frac{\partial }{\partial x}\Psi \right)\right)-kC_{\text{H}}C_{\text{B}}+S\left(x\right),$$

(B.2) $$\frac{\partial }{\partial x}\left(u\left(x\right)C_{\text{B}}\right)=\frac{1}{\theta_{s}\left(x\right)}\frac{\partial }{\partial x}\left(\theta_{s}\left(x\right)\left(D_{\text{B}}\frac{\partial }{\partial x}C_{\text{B}}+z_{\text{B}}D_{\text{B}}C_{\text{B}}\frac{\partial }{\partial x}\Psi \right)\right)-kC_{\text{H}}C_{\text{B}},$$

(B.3) $$\frac{\partial }{\partial x}\left(u\left(x\right)C_{\text{A}}\right)=\frac{1}{\theta_{s}\left(x\right)}\frac{\partial }{\partial x}\left(\theta_{s}\left(x\right)\left(D_{\text{A}}\frac{\partial }{\partial x}C_{\text{A}}+z_{\text{H}}D_{\text{A}}C_{\text{A}}\frac{\partial }{\partial x}\Psi \right)\right)+S\left(x\right),$$

(B.4) $$\frac{\partial }{\partial x}\left(u\left(x\right)C_{\text{I}}\right)=\frac{1}{\theta_{s}\left(x\right)}\frac{\partial }{\partial x}\left(\theta_{s}\left(x\right)\left(D_{\text{I}}\frac{\partial }{\partial x}C_{\text{I}}+z_{\text{I}}D_{\text{I}}C_{\text{I}}\frac{\partial }{\partial x}\Psi \right)\right),$$

(B.5) $$z_{\text{H}}C_{\text{H}}+z_{\text{B}}C_{\text{B}}+z_{\text{A}}C_{\text{A}}+z_{\text{I}}C_{\text{I}}=0.$$

Unfortunately, Eqs. (B.1) to (B.4) all contain a similar quadratic non-linearity in the electrodiffusive flux term (the product of concentration and electric potential gradient), while Eqs. (B.1) and (B.2) contain a second quadratic non-linearity in the hydrogen buffering term. For this reason, we solve the steady state equations using an iterative relaxation scheme. Given a guess for the solutions $C_{i}^{*}$ (*i* = H, B, A, I) and *Ψ**, we define the *n*-th iterates $C_{i}^{n}$ (*i* = H, B, A, I) and *Ψ*^*η*^ via the linearized equations

(B.6) $$\frac{\partial }{\partial x}\left(u\left(x\right)C_{\text{H}}^{n}\right)=\frac{1}{\theta_{s}\left(x\right)}\frac{\partial }{\partial x}\left(\theta_{s}\left(x\right)\left(D_{\text{H}}\frac{\partial }{\partial x}C_{\text{H}}^{n}+z_{\text{H}}D_{\text{H}}C_{\text{H}}^{*}\frac{\partial }{\partial x}\Psi^{n}\right)\right)-kC_{\text{H}}^{n}C_{\text{B}}^{*}+S\left(x\right),$$

(B.7) $$\frac{\partial }{\partial x}\left(u\left(x\right)C_{\text{B}}^{n}\right)=\frac{1}{\theta_{s}\left(x\right)}\frac{\partial }{\partial x}\left(\theta_{s}\left(x\right)\left(D_{\text{B}}\frac{\partial }{\partial x}C_{\text{B}}^{n}+z_{\text{B}}D_{\text{B}}C_{\text{B}}^{*}\frac{\partial }{\partial x}\Psi^{n}\right)\right)-kC_{\text{H}}^{*}C_{\text{B}}^{n},$$

(B.8) $$\frac{\partial }{\partial x}\left(u\left(x\right)C_{\text{A}}^{n}\right)=\frac{1}{\theta_{s}\left(x\right)}\frac{\partial }{\partial x}\left(\theta_{s}\left(x\right)\left(D_{\text{A}}\frac{\partial }{\partial x}C_{\text{A}}^{n}+z_{\text{H}}D_{\text{A}}C_{\text{A}}^{*}\frac{\partial }{\partial x}\Psi^{n}\right)\right)+S\left(x\right),$$

(B.9) $$\frac{\partial }{\partial x}\left(u\left(x\right)C_{\text{I}}^{n}\right)=\frac{1}{\theta_{s}\left(x\right)}\frac{\partial }{\partial x}\left(\theta_{s}\left(x\right)\left(D_{\text{I}}\frac{\partial }{\partial x}C_{\text{I}}^{n}+z_{\text{I}}D_{\text{I}}C_{\text{I}}^{*}\frac{\partial }{\partial x}\Psi^{n}\right)\right),$$

(B.10) $$z_{\text{H}}C_{\text{H}}^{n}+z_{\text{B}}C_{\text{B}}^{n}+z_{\text{A}}C_{\text{A}}^{n}+z_{\text{I}}C_{\text{I}}^{n}=0.$$

Following the same line of thinking, the linearized boundary conditions for Eqs. (B.6) to (B.9) are given by

(B.11) $$-D_{\text{H}}\frac{\partial }{\partial x}C_{\text{H}}^{n}-D_{\text{H}}z_{\text{H}}C_{\text{H}}^{*}\frac{\partial }{\partial x}\Psi^{n}+{C_{\text{H}}^{n}u|}_{x=0}={k_{\text{HI}}\left(C_{\text{I}}^{n}-\delta_{\text{HI}}C_{\text{H}}^{n}\right)|}_{x=0},$$

(B.12) $$-D_{\text{B}}\frac{\partial }{\partial x}C_{\text{B}}^{n}-D_{\text{B}}z_{\text{B}}C_{\text{B}}^{*}\frac{\partial }{\partial x}\Psi^{n}+{C_{\text{B}}^{n}u|}_{x=0}={k_{\text{AB}}\left(C_{\text{A}}-\delta_{\text{AB}}C_{\text{B}}\right)|}_{x=0},$$

(B.13) $$-D_{\text{A}}\frac{\partial }{\partial x}C_{\text{A}}^{n}-D_{\text{A}}z_{\text{A}}C_{\text{A}}^{*}\frac{\partial }{\partial x}\Psi^{n}+{C_{\text{A}}^{n}u|}_{x=0}=-{k_{\text{AB}}\left(C_{\text{A}}-\delta_{\text{AB}}C_{\text{B}}\right)|}_{x=0},$$

(B.14) $$-D_{\text{I}}\frac{\partial }{\partial x}C_{\text{I}}^{n}-D_{\text{I}}z_{\text{I}}C_{\text{I}}^{*}\frac{\partial }{\partial x}\Psi^{n}+{C_{\text{I}}^{n}u|}_{x=0}=-{k_{\text{HI}}\left(C_{\text{I}}^{n}-\delta_{\text{HI}}C_{\text{H}}^{n}\right)|}_{x=0},$$

and

(B.15) $${C_{\text{H}}^{n}|}_{x=L}=H_{L},\;{C_{\text{I}}^{n}|}_{x=L}=I_{L},$$

(B.16) $${C_{\text{B}}^{n}|}_{x=L}=B_{L},\;{C_{\text{A}}^{n}|}_{x=L}=A_{L}.$$

Solving Eqs. (B.6) to (B.10) subject to boundary conditions given by Eqs. (B.11) to (B.16) determines the *n*-th iterate for each concentration and the electric potential. We then update $C_{i}^{*}$ to be equal to $C_{i}^{n}$ and repeat. This process converges when solving the system yields new iterates which are equal to the previous ($C_{i}^{n}=C_{i}^{*}$), which gives a solution to the non-linear steady state problem Eqs. (B.1) to (B.5).

### C Spatial Discretization

Here we outline the discretization that is utilized to approximate the system of Eqs. (B.6) to (B.10). Our discretization is derived from a standard finite difference and finite volume schemes. We begin by discretizing space using a so-called “staggered grid”. Two collections of spatial points are defined, and various quantities more naturally “live” at each. The first collection of points we refer to as “cell edges,” and they are defined by

(C.17) $$x_{j}=j\;\Delta x\;j=0,1,\ldots \;N$$

where *Δx* = *L/N* is the spatial resolution of the grid. The second collection of points are referred to as “cell centers” and are defined by

(C.18) $$x_{j}=j\;\Delta x\;j=\frac{-1}{2},\frac{1}{2},\frac{3}{2}\ldots \;N+\frac{1}{2}.$$

There are in total *N* + 1 cell edges and *N* + 2 cell centers, but not all correspond to spatial locations within our domain. The cell edges *x*_0_ and *x_N_*, as well as the cell centers *x*_1/2_ and *x*_*N*+1/2_ lie either at or outside the boundary of the computational domain. These are often called “ghost points”, and quantities located at them are a numerical convenience used to help enforce boundary conditions but may not necessarily correspond to a physical quantity. All other points lie within the domain and will be referred to as interior cell centers and interior cell edges. A schematic of the spatial discretization is shown in Fig. C.4.

We approximate ionic concentrations at cell centers. Where necessary, we use a second subscript *j* to denote the spatial location where an approximation takes place, while a superscript *n* denotes *n*-th iterate of our relaxation scheme.

(C.19) $$C_{\text{i},j}^{n}\approx C_{i}^{n}\left(x_{j}\right),\;i=\text{H},\text{B},\text{A},\text{I}.$$

Finally, we introduce the quantity *Φ* to approximate the electric potential gradient. This quantity “lives” at cell edges (*j* = 0, 1, … *N*).

(C.20) $$\Phi_{j}^{n}\approx \nabla \Psi^{n}\left(x_{j}\right).$$

To approximate the Nernst-Planck type equation at interior cell centers, we use standard finite-difference and finite volume discretizations for all linear terms. The advective flux is treated with a standard upwinding scheme, while the diffusive flux is treated with a standard second order, variable coefficient, finite difference scheme [43]. In the interest of notational compactness, we utilize similar notation to denote the solvent velocity (*u*(*x*)) and volume fraction (*θ_s_*(*x*)) at various spatial locations, though no approximation is necessary as we can simply evaluate the given functions.

We are now able to discretize Eqs. (B.6) to (B.9) for each of the four ionic species, at each interior cell center (*j* = 1/2, 3/2, … *N* — 1/2):

(C.21) $$\frac{u_{j+1/2}C_{\text{H},j}^{n}-u_{j-1/2}C_{\text{H},j-1}^{n}}{\Delta x}=\frac{D_{\text{H}}}{\theta_{s,j}\Delta x^{2}}\left(\left(\theta_{s,j-1/2}\right)C_{\text{H},j-1}^{n}-\left(\theta_{s,j-1/2}+\theta_{s,j+1/2}\right)C_{\text{H},j}^{n}+\left(\theta_{s,j+1/2}\right)C_{\text{H},j+1}^{n}\right)\;+\frac{D_{\text{H}}z_{\text{H}}}{\theta_{s,j}\Delta x}\left(\theta_{s,j+1/2}\left(\frac{C_{\text{H},j+1}^{*}+C_{\text{H},j}^{*}}{2}\right)\Phi_{j+1/2}^{n}-\theta_{s,j-1/2}\left(\frac{C_{\text{H},j}^{*}+C_{\text{H},j-1}^{*}}{2}\right){\text{Φ}}_{j-1/2}^{n}\right)+S\left(x_{j}\right)-kC_{\text{H},j}^{n}C_{B,j}^{*},$$

(C.22) $$\frac{u_{j+1/2}C_{\text{B},j}^{n}-u_{j-1/2}C_{\text{B},j-1}^{n}}{\Delta x}=\frac{D_{\text{B}}}{\theta_{s,j}\Delta x^{2}}\left(\left(\theta_{s,j-1/2}\right)C_{\text{B},j-1}^{n}-\left(\theta_{s,j-1/2}+\theta_{s,j+1/2}\right)C_{\text{B},j}^{n}+\left(\theta_{s,j+1/2}\right)C_{\text{B},j+1}^{n}\right)\;+\frac{D_{\text{B}}z_{\text{B}}}{\theta_{s,j}\Delta x}\left(\theta_{s,j+1/2}\left(\frac{C_{\text{B},j+1}^{*}+C_{\text{B},j}^{*}}{2}\right)\Phi_{j+1/2}^{n}-\theta_{s,j-1/2}\left(\frac{C_{\text{B},j}^{*}+C_{\text{B},j-1}^{*}}{2}\right)\Phi_{j-1/2}^{n}\right)-kC_{\text{B},j}^{n}C_{\text{H},j}^{*},$$

(C.23) $$\frac{u_{j+1/2}C_{\text{A},j}^{n}-u_{j-1/2}C_{\text{A},j-1}^{n}}{\Delta x}=\frac{D_{\text{A}}}{\theta_{\text{s},j}\Delta x^{2}}\left(\left(\theta_{s,j-1/2}\right)C_{\text{A},j-1}^{n}-\left(\theta_{s,j-1/2}+\theta_{s,j+1/2}\right)C_{\text{A},j}^{n}+\left(\theta_{s,j+1/2}\right)C_{\text{A},j+1}^{n}\right)\;+\frac{D_{\text{A}}z_{\text{A}}}{\theta_{s,j}\Delta x}\left(\theta_{s,j+1/2}\left(\frac{C_{\text{A},j+1}^{*}+C_{\text{A},j}^{*}}{2}\right)\Phi_{j+1/2}^{n}-\theta_{s,j-1/2}\left(\frac{C_{\text{A},j}^{*}+C_{\text{A},j-1}^{*}}{2}\right)\Phi_{j-1/2}^{n}\right)+S\left(x_{j}\right),$$

(C.24) $$\frac{u_{j+1/2}C_{\text{I},j}^{n}-u_{j-1/2}C_{\text{I},j-1}^{n}}{\Delta x}=\frac{D_{\text{I}}}{\theta_{s,j}\Delta x^{2}}\left(\left(\theta_{s,j-1/2}\right)C_{\text{I},j-1}^{n}-\left(\theta_{s,j-1/2}+\theta_{s,j+1/2}\right)C_{\text{I},j}^{n}+\left(\theta_{s,j+1/2}\right)C_{\text{I},j+1}^{n}\right)\;+\frac{D_{\text{I}}z_{\text{I}}}{\theta_{s,j}\Delta x}\left(\theta_{s,j+1/2}\left(\frac{C_{\text{I},j+1}^{*}+C_{\text{I},j}^{*}}{2}\right)\Phi_{j+1/2}^{n}-\theta_{s,j-1/2}\left(\frac{C_{\text{I},j}^{*}+C_{\text{I},j-1}^{*}}{2}\right)\Phi_{j-1/2}^{n}\right).$$

We also discretize the boundary conditions for each species in a similar manner. To approximate ionic species *at* a boundary, we utilize linear interpolation (in space) using ghost points and the first interior cell center. At the left boundary of our domain (where *j* = 0) we have the following equations:

(C.25) $$k_{\text{HI}}\left(\frac{C_{\text{I},-1/2}^{n}+C_{\text{I},1/2}^{n}}{2}-\delta_{\text{HI}}\frac{C_{\text{H},-1/2}^{n}+C_{\text{H},1/2}^{n}}{2}\right)=-\frac{D_{\text{H}}}{\Delta x}\left(C_{\text{H},1/2}^{n}-C_{\text{H},-1/2}^{n}\right)+u_{0}C_{\text{H},-1/2}^{n}-D_{\text{H}}z_{\text{H}}\left(\frac{C_{\text{H},-1/2}^{*}+C_{\text{H},1/2}^{*}}{2}\right){\text{Φ}}_{0}^{n},$$

(C.26) $$-k_{\text{HI}}\left(\frac{C_{\text{I},-1/2}^{n}+C_{\text{I},1/2}^{n}}{2}-\delta_{\text{HI}}\frac{C_{\text{H},-1/2}^{n}+C_{\text{H},1/2}^{n}}{2}\right)=-\frac{D_{\text{I}}}{\Delta x}\left(C_{\text{I},1/2}^{n}-C_{\text{I},-1/2}^{n}\right)+u_{0}C_{\text{I},-1/2}^{n}-D_{\text{I}}z_{\text{I}}\left(\frac{C_{\text{I},-1/2}^{*}+C_{\text{I},1/2}^{*}}{2}\right){\text{Φ}}_{0}^{n},$$

(C.27) $$k_{\text{AB}}\left(\frac{C_{\text{A},-1/2}^{n}+C_{\text{A},1/2}^{n}}{2}-\delta_{\text{AB}}\frac{C_{\text{B},-1/2}^{n}+C_{\text{B},1/2}^{n}}{2}\right)=-\frac{D_{\text{B}}}{\Delta x}\left(C_{\text{B},1/2}^{n}-C_{\text{B},-1/2}^{n}\right)+u_{0}C_{\text{B},-1/2}^{n}-D_{\text{B}}z_{\text{B}}\left(\frac{C_{\text{B},-1/2}^{*}+C_{\text{B},1/2}^{*}}{2}\right){\text{Φ}}_{0}^{n},$$

(C.28) $$-k_{\text{AB}}\left(\frac{C_{\text{A},-1/2}^{n}+C_{\text{A},1/2}^{n}}{2}-\delta_{\text{AB}}\frac{C_{B,-1/2}^{n}+C_{B,1/2}^{n}}{2}\right)=-\frac{D_{\text{A}}}{\Delta x}\left(C_{\text{A},1/2}^{n}-C_{\text{A},-1/2}^{n}\right)+u_{0}C_{\text{A},-1/2}^{n}-D_{\text{A}}z_{\text{A}}\left(\frac{C_{\text{A},-1/2}^{*}+C_{\text{A},1/2}^{*}}{2}\right){\text{Φ}}_{0}^{n}.$$

The boundary conditions at the right are significantly simpler:

(C.29) $$\frac{C_{\text{H},N-1/2}^{n}+C_{\text{H},N+1/2}^{n}}{2}=H_{L},$$

(C.30) $$\frac{C_{B,N-1/2}^{n}+C_{\text{B},N+1/2}^{n}}{2}=B_{L},$$

(C.31) $$\frac{C_{\text{A},N-1/2}^{n}+C_{\text{A},N+1/2}^{n}}{2}=A_{L},$$

(C.32) $$\frac{C_{\text{I},N-1/2}^{n}+C_{\text{I},N+1/2}^{n}}{2}=I_{L},$$

Finally, we have a set of discrete equations which enforce the electro-neutrality constraint at the the interior cell centers (*j* = 1/2, 3/2,… *N*—1/2) *as well as the ghost point corresponding to j* = −1/2.

(C.33) $$z_{H}C_{\text{H},j}^{n}+z_{B}C_{\text{B},j}^{n}+z_{A}C_{\text{A},j}^{n}+z_{I}C_{\text{I},j}^{n}=0,\;j=-1/2,1/2,\ldots \;N-1/2.$$

We enforce the electro-neutrality constraint at the left most ghost point even though the concentrations at this point do not represent physical quantities. This is done to ensure that (up to linear approximation), the electro-neutrality constraint is satisfied up to and including the left boundary. We do not need a similar equation at the right boundary because Eqs. (C.29) to (C.32) together with choosing *H_L_, B_L_, A_L_* and *I_L_* in a manner satisfying electro-neturality implies this condition is already met.

Now, from a previous iteration (or an initial guess), we know the concentrations $C_{i,j}^{*}$, for *j* = –1/2, 1/2, … *N* + 1/2 and *i* = H, I, A, B. This implies that eqs. (C.21) to (C.33) represent a 5*N* + 9 × 5*N* + 9 system of linear equations which may be solved for concentrations and the potential gradient (simultaneously) at the next iteration ($C_{i,j}^{n}$ and $\phi_{j}^{n}$).

#### C.1 Convergence Criteria

To calculate the steady state ion concentrations, the numerical discretization and relaxation scheme outlined above are iterated. This produces a sequence of approximate ion concentrations

$$C_{i,j}^{1},C_{i,j}^{2},\ldots \;C_{i,j}^{n}.$$

After *n* iterations, the error associated with ion *i* is approximated by

(C.34) $$E_{i}\left(n\right)=\underset{j=-1/2,1/2,\ldots \;N+1/2}{\max}\left|C_{i,j}^{n}-C_{i,j}^{n-1}\right|.$$

We stop iteration when the maximum error (over all ion species) is less than a specified tolerace

(C.35) $$\underset{i=\text{H},\text{I},\text{B},\text{A}}{\text{max}}E_{i}\left(n\right)<\delta .$$

In the results reported here, we used a tolerance of *δ* = 5 × 10^−10^.

### D Calculation of Sobol Indices

Using the notation from [39], we begin with generating two independent sampling matrices **A** and **B**, each of size *s* × *N*, where *s* is the number of samples and *N* is the number of parameters. Hence, each row represents a sample and each column represents a parameter. We define a matrix ${\text{A}}_{\text{B}}^{\left(i\right)}$ where all columns are from **A** except the *i*-th column which is from **B**. Then, the total Sobol’ index for the parameter *p_i_* is estimated by,

(D.36) $${\hat{\bar{S}}}_{i}=\frac{1}{2s}\overset{s}{\underset{j=1}{\sum }}{\left(f{\left(A\right)}_{j}-f{\left(A_{B}^{\left(i\right)}\right)}_{j}\right)}^{2},$$

where, *f*(**A**)_*j*_ is the QoI for the parameters corresponding to the *j*-th row of the matrix **A** and $f{\left(A_{B}^{\left(i\right)}\right)}_{j}$ is the QoI for the parameters corresponding to the *j*-th row of the matrix $A_{B}^{\left(i\right)}$.

To estimate the first-order SI, we implemented the “Correlation 2” scheme proposed in [41]. This scheme uses three independent sampling matrices **A, B**, and **C**. Using the above notation, the estimator is defined by,

(D.37) $$\hat{{\underline{S}}_{i}}=\frac{1}{s}\overset{s}{\underset{j=1}{\sum }}\left(f{\left(A\right)}_{j}-f{\left(A_{C}^{\left(i\right)}\right)}_{j}\right)\left(f{\left(B_{A}^{\left(i\right)}\right)}_{j}-f{\left(B\right)}_{j}\right),$$

where, $f{\left(A_{C}^{\left(i\right)}\right)}_{j}$ is the QoI for the parameters corresponding to the *j*-th row of the matrix $A_{C}^{\left(i\right)}$. Following our terminology, $A_{C}^{\left(i\right)}$ is the matrix where all columns are from **A** except the *i*-th column, which is from the third independent matrix **C**. To sample the parameter space we use Sobol’ sequences, which are quasi-random sequences that have been shown to give a uniform distribution with low discrepancy in high-dimensional spaces [44]. To reduce the computation time of the QoI for a sample, custom sparse arrays based on compressed sparse row format were developed to represent the discretized equations (Eqs. (C.21) to (C.33)). The resulting system of equations was solved using the sparse linear solver scipy.sparse.linalg.spsolve (with permc_spec=MMD_AT_PLUS_A). Further, rather than using a static number of samples for the estimation of SI, we define a convergence criterion to dynamically select the number of samples to estimate the SI of each parameter.

The estimated SI is updated with every new sample in the parameter space. This gives us a statistical distribution of the updates of the estimated SI. We define the convergence of SI if the standard deviation of this distribution, over the last *k* updates (we call this the batch size), is less than a tolerance tol. If the model is well-behaved in the parameter regime, we expect the estimated SIs to converge as we include more samples. Hence, we expect that the standard deviation of the last batch of *k* number of updates of the SI will be considerably low. Figure D.5 shows the convergence of the estimated SIs for the model in this study in the full parameter regime. If we decrease tol, more number of updates are required for convergence of SI of some of the parameters, without a significant change in the estimated SI.

Finally, for faster computation, the QoIs required for the updates of an entire batch (defined by *k*) are computed in parallel using the package joblib in Python.
